# FOXO3a/miR-4259-driven LDHA expression as a key mechanism of gemcitabine sensitivity in pancreatic ductal adenocarcinoma

**DOI:** 10.1186/s40170-025-00377-3

**Published:** 2025-02-10

**Authors:** Tung-Wei Hsu, Wan-Yu Wang, Hsin-An Chen, Tzu-Hsuan Wang, Chih-Ming Su, Po-Hsiang Liao, Alvin Chen, Kuei-Yen Tsai, George Kokotos, Cheng-Chin Kuo, Ching-Feng Chiu, Yen-Hao Su

**Affiliations:** 1 Department of Surgery, Division of General Surgery, Shuang Ho Hospital, Taipei Medical University, New Taipei City, Taiwan; 2 Department of Surgery, Division of General Surgery, School of Medicine, College of Medicine, Taipei Medical University, Taipei, Taiwan; 3 TMU Research Center of Cancer Translational Medicine, Taipei Medical University, Taipei, Taiwan; 4 TMU Research Center for Digestive Medicine, Taipei Medical University, Taipei, Taiwan; 5 Department of Surgery, School of Medicine, College of Medicine, Taipei Medical University, Taipei, Taiwan; 6 Cardiovascular and Mitochondrial Related Disease Research Center, Hualien Tzu Chi Hospital, Buddhist Tzu Chi Medical Foundation, Hualien, Taiwan; 7 Department of Chemistry, National and Kapodistrian University of Athens, Panepistimiopolis, Athens, Greece; 8 Institute of Cellular and System Medicine, National Health Research Institutes, Zhunan, Taiwan; 9 Department of Bioscience Technology, Chung Yuan Christian University, Taoyuan, Taiwan; 10 Metabolic and Weight Management Center, Shuang Ho Hospital, Taipei Medical University, New Taipei City, Taiwan

**Keywords:** FOXO3a, Gemcitabine resistance, Lactate dehydrogenase A, microRNA-4259

## Abstract

**Background:**

Lactate dehydrogenase A (LDHA) can regulate tumorigenesis and cancer progression. Nevertheless, whether the regulation of LDHA is involved in the development of gemcitabine resistance in PDAC has not yet been fully elucidated. Increasing studies have shown that cancer acquired drug resistance led to treatment failure is highly attributed to the cancer stem cell (CSC) properties. Therefore, we aim to demonstrate the functions and regulatory mechanisms of LDHA on cancer stem cell (CSC) properties and gemcitabine resistance in PDAC.

**Methods:**

We investigate the metabolite profiles by liquid chromatography-mass spectrometry between gemcitabine–resistant PDAC and parental PDAC cells. Additionally, gain-of-function and loss-of-function experiments were conducted to examine the roles of LDHA on CSC properties and gemcitabine resistance in the gemcitabine–resistant PDAC and parental PDAC cells. To investigate regulators involved in LDHA-mediated gemcitabine resistance and CSC of pancreatic cancer cells, we further used a combination of the miRNA microarray results and software predictions and confirmed that miR-4259 is a direct target of LDHA by luciferase assay. Furthermore, we constructed serial miR-4259 promoter reporters and searched for response elements using the TESS 2.0/TFSEARCH software to find the transcription factor binding site in the promoter region of miR-4259.

**Results:**

We observed that elevated LDHA expression significantly correlates with recurrent pancreatic cancer patients following gemcitabine treatment and with CSC properties. We further identify that FOXO3a-induced miR-4259 directly targets the 3’untranslated region of LDHA and reduced LDHA expression, leading to decreased gemcitabine resistance and a reduction in the CSC phenotypes of pancreatic cancer.

**Conclusion:**

Our results demonstrated that LDHA plays a critical role in cancer stemness and gemcitabine resistance of pancreatic cancer, and indicate that targeting the FOXO3a/miR-4259/LDHA pathway might serve as a new treatment for pancreatic cancer patients with a poor response to gemcitabine chemotherapy.

**Supplementary Information:**

The online version contains supplementary material available at 10.1186/s40170-025-00377-3.

## Introduction

Pancreatic ductal adenocarcinoma (PDAC) comprises more than 90% of pancreatic cancers and is the most lethal solid tumour [1]. Only approximately 6% of patients with PDAC are still alive at five years due to late diagnosis, difficult resection and poor prognosis [1]. Gemcitabine is a difluoro analog of deoxycytidine that has been used as a standard chemotherapy for patients with locally advanced or metastatic PDAC for the past two decades [2]. However, gemcitabine-based treatment has shown poor therapeutic efficiency and survival outcomes due to the development of drug resistance and tolerance [3]. There is urgent need to understand the mechanism(s) underlying the gemcitabine resistance of PDAC to improve therapeutic efficiency and develop better therapeutic strategies.

Cancer has been considered to be a metabolism-related disease and metabolic reprogramming is a common feature of cancer [4]. Emerging evidence has also revealed a strong connection between metabolic dysregulation and cancer progression [5]. It has been shown that many human cancers have higher expression levels of lactate dehydrogenase (LDH), which catalyzes the conversion of pyruvate to lactate, which is accompanied by NADH oxidation and reduction, thus regulating cellular energy metabolism under anaerobic conditions as a result of the Warburg effect [6]. LDH is a homo- or hetero-tetramer assembled from LDHA and LDHB, which results in five major isoforms, numbered LDH-1 through LDH-5, and alterations in the expression or activity of LDHA directly influence the Warburg effect, severely affecting glucose metabolism [7]. Therefore, altered expression of LDHA may contribute to tumorigenesis and cancer progression. Recently, several studies have shown that the transcriptional and post-translational regulation of LDHA by KLF4, FOXM1 and lysine-5 acetylation promotes aerobic glycolysis and the progression of pancreatic cancer [8]. Additionally, when the expression of LDHA in taxol-resistant breast cancer cells was suppressed, the cells showed a better response to chemotherapy [9]. Nevertheless, whether the regulation of LDHA is involved in the development of gemcitabine resistance in PDAC has not yet been fully elucidated.

MicroRNAs (miRNAs) are a group of endogenous and small non-coding ~ 22 nt RNAs, which downregulate the post-transcriptional level of target protein(s) by binding to the 3’ untranslated region (UTR) of their mRNA. Recent studies have shown that miRNAs play important role(s) as oncogenes or tumour suppressors based on their target genes. Moreover, miRNAs not only play important regulatory roles in a variety of biological processes but also participate in controlling cancer cell metabolism by directly or indirectly modulating the expression of metabolic enzymes [10]. Increasing evidence suggests that the function of miRNAs in controlling cellular metabolism and regulating cancer stem cell (CSC) phenotypes may offer new molecular strategies for the development of novel therapeutic and synergistic treatments to overcome chemoresistance in malignant cancer [11, 12].

In this study, we uncovered a novel mechanism by which miR-4259 is transcribed by FOXO3a and suppresses LDHA expression to inhibit the gemcitabine resistance and CSC properties of pancreatic cancer. We further observed that LDHA expression inversely correlated with the levels of miR-4259 and FOXO3a in recurrent pancreatic cancer following gemcitabine treatment. These findings suggest that the suppression of LDHA by FOXO3a/miR-4259 plays a critical role in mitigating LDHA-mediated cancer stemness and overcoming gemcitabine resistance in pancreatic cancer.

## Methods

### Reagents and antibodies

Culture-related culture media, fetal bovine serum and penicillin-streptomycin were purchased from Invitrogen (Thermo Fisher Scientific Inc., Waltham, MA, USA). Gemcitabine hydrochloride (M1716) was purchased from AbMole BioScience (Houston, TX, USA). Polyethyleneimine (PEI) and polybrene were purchased from Sigma-Aldrich (St Louis, MO, USA). Protease inhibitor cocktail was purchased from Roche (Basel, CH, Switzerland). pUSEamp-myr-Akt1 (myr-AKT) was purchased from Upstate Biotechnology, Inc. (Lake Placid, NY, USA) and used to generate constitutively active AKT. The AKT inhibitor (Akt Inhibitor IV, #124015) was purchased from Calbiochem, Merck Millipore (Darmstadt, Germany) and used to inhibit AKT activity in relevant experiments. Western blotting and ChIP were performed with the following antibodies: LDHA (#A1146, dilution 1/1000, ABclonal, Woburn, MA, USA), FOXO3a (sc-11351, dilution 1/1000, Santa Cruz Biotechnology, Santa Cruz, CA, USA), PTEN (#9559, dilution 1/1000, Cell Signaling Technology), Phospho-Akt (Ser473) (#9271, dilution 1/1000, Cell Signaling Technology), AKT (#9272, dilution 1/1000, Cell Signaling Technology), Lamin B (GTX109894, dilution 1/2000, GeneTex Inc., Irvine, CA, USA) and α-tubulin (T6074, dilution 1/100000, Sigma-Aldrich). All secondary antibodies were purchased from Jackson ImmunoResearch (West Grove, PA, USA).

### PDAC cell lines and gemcitabine-resistant clones

Human pancreatic ductal adenocarcinoma (PDAC) cell lines (PANC-1, MIA PaCa-2, BxPC-3 and SUIT-2 cells) and human embryonic kidney 293 (HEK293T) cells were purchased from the American Type Culture Collection (ATCC, Manassas, VA, USA) and grown in culture medium according to their instructions. These cells were free of mycoplasma contamination and their identity was confirmed by STR profiling at the Bioresource Collection and Research Center (Hsinchu, Taiwan) and Center for Genomic Medicine, NCKU (Tainan, Taiwan). All cells were cultured in appropriate medium: RPMI-1640 medium for SUIT-2 and BxPC-3 cells, and high-glucose/DMEM medium for PANC-1, PANC-1/GEM, MIA PaCa-2, MIA PaCa-2/GEM and HEK293T cells. Each medium was supplemented with 10% fetal bovine serum and 1% penicillin/streptomycin, with 2.5% horse serum added for MIA PaCa-2 and MIA PaCa-2/GEM cells. All cells were grown at 37 °C in a humidified 5% CO_2_ atmosphere. The gemcitabine-resistant PANC-1 (PANC-1/GEM) and MIA PaCa-2 (MIA PaCa-2/GEM) cell lines were gifts from Dr. Wun-Shaing Wayne Chang and Dr. Li-Tzong Chen (National Health Research Institutes, Miaoli, Taiwan). These cell lines were maintained in culture medium supplemented with gemcitabine at a final concentration of 5 µM, which was gradually increased during their development to ensure stable resistance. To further characterize the relevance of selected cells with other PDAC cells, we examined the gemcitabine sensitivity of PDAC cell lines by MTT assay and flow cytometric analysis (Supplementary Fig. 1a, b).

### Metabolomic analyses

The approximately 80% confluent PANC-1 and PANC-1/GEM cells were cultured in 10% FBS medium for one day and the supernatant was collected after centrifuging the cells at 3,000 rpm for 10 min at 4 °C. 10 ml of the supernatant was mixed with 40 ml cold methanol by gentle shock for 30 min at 4 °C. The clear supernatant was collected, lyophilized and stored at -80 °C for further analysis. We compared the metabolite profiles of both types of cells and performed ultra-performance liquid chromatography (Acquity UPLC System, Waters Corporation, Milford, MA, USA) coupled with time-of-flight mass spectrometry (Xevo TOF MS, Waters Corporation). The quadrupole-time of flight (QTof) mass spectrometry (MS) system (QTof-MS) was operated in positive electrospray ionization mode with a mass resolution larger than 10,000. The MS data were acquired and submitted to analysis with the MarkerLynx software (Waters Corporation) to convert the raw data into exact mass − retention time pairs (EMRTs). EMRTs with *P* values < 0.05 and a factor of change greater than 1.5 were selected for further analysis. These m/z values were searched by Biomolecules database of MarkerLynx software and The Human Metabolome Database (version 3.6, online database). Mass spectrometry analyses were performed by the Research Facility for Sharing at NHRI, Taiwan (Core Facilities for Proteomics and Chemistry).

### LDH activity and lactate production assay

The LDH activity of the PDAC cells was detected using the Lactate Dehydrogenase (LDH) Activity Assay Kit (K726-500, BioVision, Inc., Milpitas, CA, USA), according to the manufacturer’s instructions. Briefly, 1 × 10^6^ cells were seeded in a 60-mm dish one day prior to the assay, which was performed in triplicate. The cells were collected and washed, and proteins were extracted to analyze the total LDH activity. Lactate production of culture medium of PDAC cells was measured by Lactate Colorimetric/Fluorometric Assay Kit (K607-100, BioVision) according to the manufacturer’s instructions.

### Plasmid constructs, shRNA clones and miRNA inhibitors

Full-length human *LDHA* (NM_005566) was amplified using the cDNA from PANC-1 cells by polymerase chain reaction (PCR) and was cloned into the pcDNA6 vector (Invitrogen) and pCDH-CMV-MCS-EF1-copGFP + Puro (pCDH) lentiviral expression vector (#CD513B-1, System Biosciences, Mountain View, CA, USA). Full-length human *FOXO3a* was amplified by PCR using the cDNA from HeLa cells and was cloned into the pcDNA6 vector and pCDH lentiviral expression vector. The stem-loop sequence of miR-4259 (Sanger Center miRNA Registry, http://microrna.sanger.ac.uk/sequences/) was amplified from the genomic DNA of PANC-1 cells by PCR and was cloned into the *XhoI* and *NotI* sites of pLemiR (Open Biosystems, Huntsville, AL, USA) and pcDNA vector. The miR-4259 promoter reported in the miRStart database (http://mirstart.mbc.nctu.edu.tw/home.php) was amplified from the genomic DNA of PANC-1 cells by PCR and was cloned into the *NheI* and *XhoI* sites of pGL3-Basic (Promega, Fitchburg, WI, USA). This was subjected to further serial deletion to identify the transcription factor binding sites predicted by PROMO database (http://alggen.lsi.upc.es/cgi-bin/promo_v3/promo/promoinit.cgi?dirDB=TF_8.3). The human full-length *LDHA* 3’UTR (NM_005566) was amplified from the genomic DNA of PANC-1 cells by PCR and was cloned into the *NheI* and *XhoI* sites of the pmirGLO Dual-Luciferase vector (Promega). The *LDHA* 3’UTR mutations of the miR-4259 binding site were amplified from the wild-type 3’UTR by PCR-driven overlap extension [13] and were subsequently cloned into the *NheI* and *XhoI* sites of pmirGLO. All primers used are listed in Supplementary Tables 7 and all plasmids were confirmed by DNA sequencing.

The serial deletion constructs of the miR-4259 promoter were further amplified and cloned as described above. The *LDHA*-3’UTR containing the mutant miR-4259 target sequences and the miR-4259 promoter containing the mutant FOXO3a binding site (FOXO3a BS) sequence and the FOXO3a mutant constructs, FOXO3a(3 A), were generated by site-directed mutagenesis using the QuickChange II XL Site-Directed Mutagenesis Kit (Stratagene, La Jolla, CA, USA). Specifically, the three AKT phosphorylation sites (Thr32, Ser253, and Ser315) were mutated to alanine (designated as FOXO3a(3 A)) to render FOXO3a constitutively active. All primer sequences of the constructs are shown in Supplementary Tables 7, and the mutation constructs were confirmed by DNA sequencing.

The lentiviral *LDHA* shRNA clones (TRCN0000026538, TRCN0000164922 and TRCN0000165175), *FOXO3a* shRNA clones (TRCN0000010334 and TRCN0000235488), pLKO.1-shLuc control clone (TRCN0000072244), the pMD2.G plasmid and the pCMVdeltaR8.91 plasmid were purchased from the National RNAi Core Facility at Academia Sinica, Taipei, Taiwan. The miR-4259 antagomir (anti-miR-4259) and control were purchased from PhalanxBio Inc. (Hsinchu, Taiwan).

### Transfection and lentivirus infection

To overexpress LDHA, 2 × 10^5^ cells were transfected with a LDHA expression plasmid or a control vector (pcDNA6) for 48 h using the TOOLSFect transfection reagent (BIOTOOLS Co., Ltd., Taipei, Taiwan) according to the manufacturer’s protocol. For lentivirus production, the envelope plasmid (pMD2.G), packaging plasmid (pCMV-deltaR8.91) and target genes (LDHA and FOXO3a shRNA clones and luciferase shRNA control clone) were transfected into HEK-293T cells using the PEI (Polyethylenimine, Sigma) reagent, according to the manufacturer’s instructions. The supernatant was collected and then filtered with a 0.2 μm syringe filter after 48 h. For lentivirus infection, 1 × 10^6^ cells were infected with 2 ml of lentivirus containing 8 µg ml^− 1^ polybrene for 24 h, then the medium was changed and the cells were incubated for another 48 h. To select and establish stable cell lines, the cells were cultured with puromycin.

### RNA isolation and quantitative RT-PCR

Total RNA was isolated using Trizol (Invitrogen) and as a template to reverse transcript into cDNA by M-MLV reverse transcript kit (Invitrogen) following the manufacturer’s instructions. Quantitative RT-PCR was performed using Lightcycler 480 system (Roche). The values of the threshold cycle (CP) were calculated using Lightcycler 480 software. The relative mRNA expression was normalized to the mean of internal *GAPDH*. The relative levels of gene expression were represented as ΔCP = CP of tested *gene*– CP of reference *gene*.

The mature miRNA sequences were obtained from the Sanger Center miRNA Registry (http://microrna.sanger.ac.uk/sequences/) and the stem-loop RT primers were designed according to Chen et al. [14]. For mature microRNA detection, real-time RT-PCR reactions contained 0.5 µM of each forward and reverse primer, 1 µM Universal ProbeLibrary Probe #21 (Roche), 1× LightCycler TaqMan Master mix, and 2 µl of cDNA. Amplification curves were generated with an initial denaturing step at 95 °C for 10 min, followed by 65 cycles of 95 °C for 5 s, 60 °C for 10 s and 72 °C for 1 s. The *U47* small nuclear RNA was used as the reference gene. The relative levels of gene expression were represented as ΔCP = CP of target *gene*– CP of reference *gene*, and the fold change of gene expression were calculated by the 2^−ΔΔCP^.

For pre-miR-4259 and pri-miR-4259 detection were determined by RT-qPCR as previously described [15]. Brifly, 1 µg of total RNA was used in reverse transcription by random hexamer primer. Transcripts were detected by quantitative PCR with the LightCycler FastStart DNA Master SYBR Green I kit (Roche). The sequences of pre-miR-4259 and pri-miR-4259 obtained from the miRbase website (http://www.mirbase.org/index.shtml) and miRStart database (http://mirstart.mbc.nctu.edu.tw/home.php), respectively and internal reference *GAPDH* are shown in Supplementary Table 7. PCR reactions contained 0.5 µM of each forward and reverse primer, 1× Master SYBR Green mix, and 2 µL of cDNA. Amplification curves were generated with an initial denaturing step at 95 °C for 10 min, followed by 55 cycles of 95 °C for 5 s, 60 °C for 10 s and 72 °C for 1 s. A dissociation procedure was performed to generate a melting curve for confirmation of amplification specificity. The relative expression levels of pri-miR-4259 and pre-miR-4259 were calculated using 2^−(CP of pri−miR−4259– CP of *GAPDH* gene)^ and 2^−(CP of (pri−miR−4259 + pre−miR−4259)– CP of pri−miR−4259)^, respectively.

### Cellular fractionation

The cytosolic and nuclear fractions were extracted as previously described [16]. Briefly, cells were washed twice with ice-cold PBS, harvested by scraping with a rubber policeman, and lysed in buffer A (20 mM HEPES, pH 7.0, 10 mM KCl, 2 mM MgCl_2_, 0.5% NP-40, 1 mM Na_3_VO_4_, 10 mM NaF) containing protease inhibitor cocktail (Roche). After 10 min incubation on ice, cells were homogenized by 15–20 strokes in a tightly fitting Dounce homogenizer and centrifuged 5 min at 1,500*g* to sediment the nuclei. The supernatant is the cytosolic fraction. To remove contamination from cytoplasmic membranes, the nuclear pellet was washed ten times with buffer A. To extract nuclear proteins, the isolated nuclei were resuspended in NETN lysis buffer (20 mM Tris-HCl, pH 8.0, 150 mM NaCl, 0.5% Nonidet P-40 and 1 mM EDTA) containing protease inhibitor cocktail (Roche) and the mixture was sonicated briefly to aid nuclear lysis. After centrifugation at 16,100*g* for 20 min at 4 °C the nuclear lysates were collected. Cytosolic fraction and nuclear fractions were analysed by Western blot.

### Sphere formation assay

Cells were dissociated with trypsin-EDTA and the resulting single cells were centrifuged to remove the enzymes and re-suspended in serum-free medium containing B27 supplement (Invitrogen), 20 ng ml^− 1^ EGF (Invitrogen) and 10 ng ml^− 1^ bFGF (Invitrogen) and were allowed to re-form sphere-like cells. A 1,000 cells/well diluted cell suspension was plated onto ultra-low attachment 24-well plates (Corning Inc., Corning, NY, USA) for seven to fourteen days. The results are representative of three independent experiments.

### Flow cytometric analysis

For studing the gemcitabine-induced cell death, gemcitabine treatment was performed under serum-free conditions to minimize potential interference from serum components, providing a controlled environment to study the intrinsic cellular response and CSC-related phenotypes during drug treatment. PDAC cells were incubated with serum-free medium with 4 µM gemcitabine for 48 h. Aliquots of 2 × 10^5^ cells were collected and washed twice with ice-cold PBS and then fixed with ice-cold 70% ethanol overnight. After fixation, cells were washed with PBS to remove residual ethanol, pelleted, and resuspended in PBS containing 50 µg ml^− 1^ of propidium iodide (PI; Sigma-Aldrich) and 10 µg ml^− 1^ of RNaseA (R4642, Sigma-Aldrich). Staining was done at 4 °C for at least 30 min, and samples were analyzed using a flow cytometry (FACSCalibur; BD Biosciences, San Jose, CA, USA). For stem cell markers analysis, 5 × 10^5^ of PDAC cells were harvested and washed with ice-cold PBS. Cell pellets were resuspended in 50 µl of PBS and then incubated with a 1:50 dilution of anti-CD133 antibody (PE, 130-080-801, Miltenyi Biotech, Bergisch Gladbach, Germany) for 30 min at 4 °C. Those cells were washed by ice-cold PBS, resuspended in 0.5 ml ice-cold PBS and subsequent analysis by flow cytometry (FACSCalibur). Nonspecific mouse IgG antibody was used as isotype control for comparison (Supplementary Fig. 3e). ALDH enzymatic activity of PDAC cells was determined by Aldefluor kit (StemCell Technologies, Vancouver, BC, Canada) according to the manufacturer’s protocol and previous studies [15]. Brightly fluorescent ALDH-positive cells were detected by flow cytometry analysis and samples treated with the specific ALDH inhibitor, diethylaminobenzaldehyde (DEAB), were used as the control to set the gates defining the ALDH-positive region (Supplementary Fig. 3f). Results are representative of three independent experiments.

### miRNA microarray hybridization

Five micrograms of total RNA obtained from PANC-1 and PANC-1/GEM cells were labeled and hybridized on miRNA microarrays (using the Human miRNA OneArray^®^ v2 (Phalanx Biotech Group, San Diego, CA, USA). The arrays were designed to detect the 1,087 unique miRNA probes from humans (miRBase Release, http://www.mirbase.org/). The miRNAs that were significantly upregulated or downregulated were determined using the GenePix 4.1 software (Molecular Devices, Silicon Valley, CA, USA). Microarray data have been deposited in the Gene Expression Omnibus (GEO) database: GSE79234.

### Western blotting

Cells were washed twice with PBS, lysed in NETN lysis buffer (20 mM Tris-HCl, pH 8.0, 150 mM NaCl, 0.5% Nonidet P-40 and 1 mM EDTA) containing protease inhibitor cocktail for 5 min with sonication and then centrifuged at 14,000×g for 30 min. An equal quantity of protein from cell lysates was resuspended in gel sample buffer, resolved by SDS-polyacrylamide gel electrophoresis, and transferred to PVDF membranes (Millipore). After blocking, blots were incubated with specific primary antibodies, and after washing and incubating with secondary antibodies, immunoreactive proteins were visualized using an enhanced chemiluminescence detection system (PerkinElmer, Waltham, MA, USA). All uncropped blots are shown in Supplementary Fig. 10.

### *LDHA* 3’UTR and miR-4259 promoter reporter assay

For the *LDHA*-3’UTR reporter assay, the indicated ratios of *LDHA*-3’UTR reporter constructs and miR-4259 expression vectors were co-transfected into HEK293T cells (50% confluent in 24-well plates) using PEI (Sigma-Aldrich) according to the manufacturer’s instructions. For the miR-4259 promoter reporter assay, HEK293T cells were transfected with miR-4259 promoter reporter constructs, plus pRL-TK and either FOXO3a or pcDNA plasmids. All cell extracts were prepared 48 h after transfection, and luciferase activities were determined by the Dual-Luciferase Reporter Assay System (Promega) following the protocols provided by manufacturer. Luciferase activity was assessed by normalization of firefly luciferase activity to Renilla luciferase activity.

### Chromatin immunoprecipitation assay

The chromatin immunoprecipitation assay was performed with the EZ ChIP kit (Millipore) as previously described [17]. Briefly, cells were fixed with 1% formaldehyde, washed and lysed. The cell lysates were sonicated to shear DNA to sizes of 300-1,000 bp. Protein-DNA complexes were precipitated with either normal rabbit IgG (sc-2027 X, Santa Cruz Biotechnology) or target protein FOXO3a (sc-11351 X, Santa Cruz Biotechnology) overnight at 4 °C with rotation. After reverse cross-link of protein-DNA complexes to free DNA, qPCR was performed with the LightCycler 480 (Roche) by using LightCycler FastStart DNA Master SYBR Green I kit (Roche) and specific primers (Supplementary Table 7). Cycling conditions were 95 °C for 10 min followed by 50 cycles of 95 °C for 15 s, 60 °C for 1 min. A dissociation procedure was performed to generate a melting curve for confirmation of amplification specificity. The relative occupancy of the immunoprecipitated factor at a locus was estimated by the comparative threshold method [18].

### Specimens

Patients with pancreatic cancer who underwent surgical resection followed by gemcitabine-based adjuvant chemotherapy at Taipei Medical University Shuang-Ho Hospital between June 2002 and August 2019 were included in this study. The retrospective study has been approved by the Institutional Review Board of academic institute. A total of 20 patients with stage I, II, and III pancreatic adenocarcinomas were included, and each patient received follow-up and imaging studies by hospital practice guidelines usually at 3-month intervals. The cut-off date for the analysis was December 2019. The recurrence-free survival rate was defined as the period from surgery until tumour recurrence death which even came first.

Total RNA was extracted from whole pancreatic tumour tissues using a High Pure FFPE RNA Micro Kit (Roche), following the manufacturer’s instructions. Reverse transcription was carried out at 42 °C for 90 min and 95 °C for 5 min, followed by incubation at 72 °C for 15 min using 10 µg total RNA, random hexamer primers (Roche Applied Science), and M-MLV reverse transcriptase (Invitrogen). RT-qPCR was performed using the Lightcycler 480 system (Roche). The values of the threshold cycle (CP) were calculated using the Lightcycler 480 software. The relative mRNA and miRNAs levels of the target genes were normalized to the means of the internal controls (*GAPDH* and *U47*, respectively). The relative levels of gene expression were represented as ΔCP = CP of the tested *gene*– CP of the reference *gene*. Lower ΔCP values indicate higher expression of the *gene*. The median of individual ΔCP values of patient samples was used as cut-off values to define high and low expression.

### Animal studies

All animal work was conducted in accordance with a protocol approved by the Institutional Animal Care and Use Committee of the National Health Research Institutes (NHRI-IACUC-104045-AP). Male NOD/SCID (NOD.CB17-*Prkdc*^*scid*^/NcrCrl) or ASID (advanced severe immuno-deficiency, NOD.Cg-*Prkdc*^*scid*^*Il2rg*^*tm1Wjl*^*/*YckNarl) mice, aged 4–6 weeks, were purchased from the National Laboratory Animal Center, Taipei, Taiwan. Tumorigenic experiments were conducted using human pancreatic ductal adenocarcinoma (PDAC) cell lines, including PANC-1 (parental) and PANC-1/GEM (gemcitabine-resistant) cells, as well as MIA PaCa-2 and MIA PaCa-2/GEM cells (used in supplementary experiments). Modifications to these cells included overexpression or suppression of FOXO3a (via lentiviral vectors or shRNA), miR-4259 modulation (overexpression using lentiviral vectors or suppression using antagomiRs), and LDHA modulation (knockdown using shRNA or overexpression via lentiviral constructs), with appropriate control groups using empty vectors or scrambled constructs. For tumorigenesis experiments, 5 × 10⁶ modified cells mixed with Matrigel were subcutaneously injected into the dorsal flanks of mice. Tumor volumes were measured every three days using calipers and calculated as 1/2 (length × width²). When tumors reached 100–200 mm³, mice were treated with intraperitoneal injections of gemcitabine (50 mg/kg, once per week) or vehicle control. Tumor tissues were collected post-mortem for histological, biochemical, and molecular analyses, including Western blot, RT-qPCR, and immunohistochemistry to assess FOXO3a, miR-4259, and LDHA expression. To assess tumor-initiating capacity, serially diluted PDAC cells (ranging from 1 × 10⁵ to 1 × 10² cells) were injected into ASID mice, with tumor initiation monitored over 49 days and tumor-initiating cell (TIC) frequency calculated using L-Calc software (StemCell Technologies) [19].

### Statistics

All statistical analyses were performed with the Prism 6 software (La Jolla, CA, USA). Data from the in vitro experiments were approximately normally distributed and are presented as the means ± s.e.m. from at least three independent experiments, each of which was performed in triplicate. The statistical evaluation of variance among the experimental groups was similar based on a two-tailed Student’s *t*-test for comparisons between two groups. Recurrence-free survival outcomes were assessed using the Kaplan-Meier method and log-rank test, and correlations were calculated using Pearson’s test (two-tailed). A *P*-value < 0.05 was considered to indicate significance.

### Data Availability

The microarray data (GSE79234) referenced during the study are available in a public repository from the Gene Expression Omnibus (GEO) website (https://www.ncbi.nlm.nih.gov/geo/). The authors declare that all the other data supporting the findings of this study are available within the article and its supplementary information files and from the corresponding author upon reasonable request.

## Results

### LDHA expression is critical for gemcitabine resistance

**Fig. 1:**
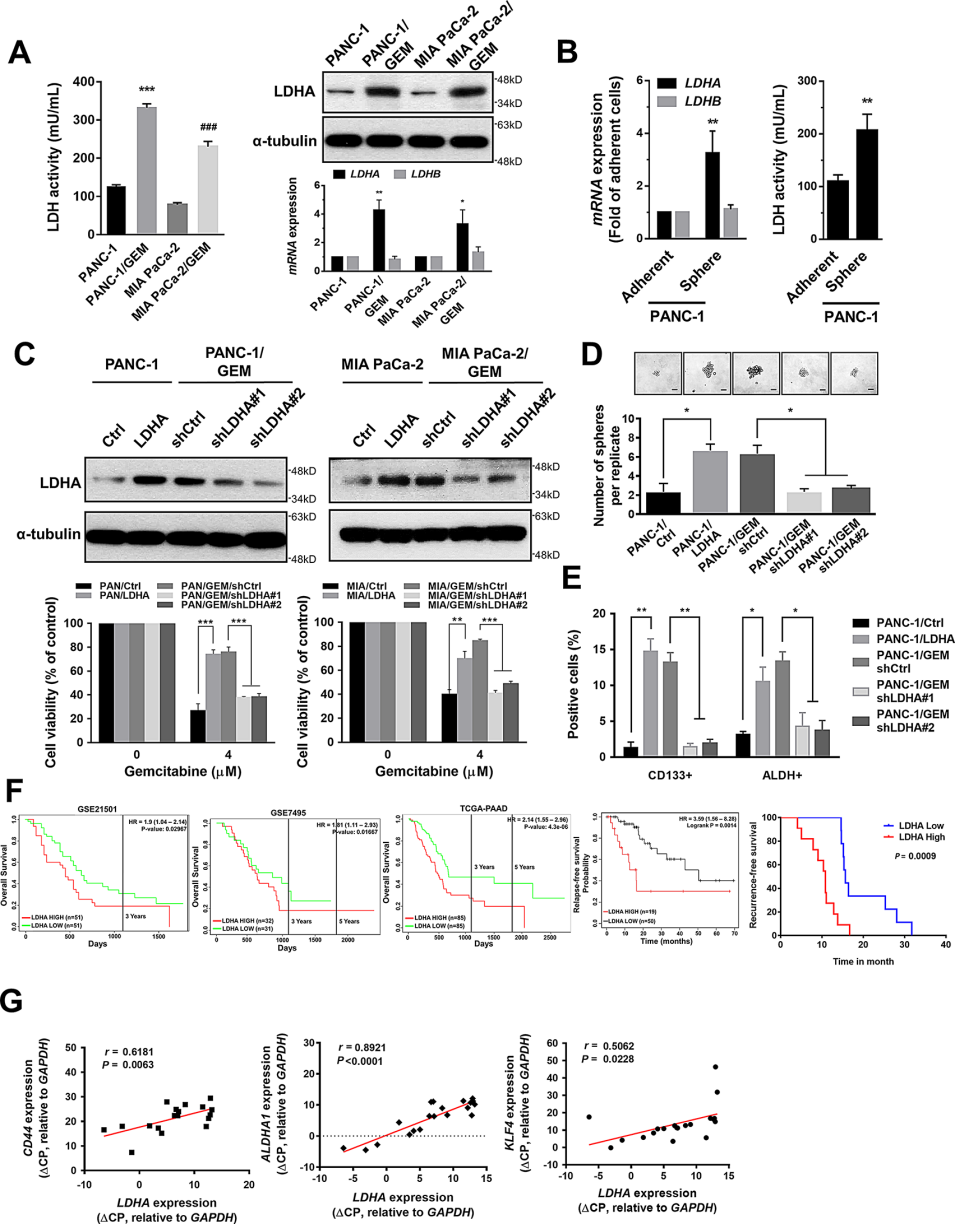
LDHA enhances the gemcitabine resistance and CSC properties of PDAC cells. (**A**) Left panel, the LDH enzyme activity in the PANC-1, PANC-1/GEM, MIA PaCa-2 and MIA PaCa-2/GEM cells. Right panel, the mRNA expression of *LDHA* and *LDHB* (*bottom*) and the protein expression of LDHA (top) were measured by RT-qPCR and Western blotting in these PDAC cell lines. α-tubulin was used as the internal protein loading control. (**B**) The *LDHA* and *LDHB* expression (left), and LDH enzyme activity (right) were determined for adherent and sphere PANC-1 cells. (**C**) Top, the protein expression of LDHA was analyzed by Western blotting in the indicated cell lines. *Bottom*, the viability of the indicated cell lines was measured by the MTT assay after treatment with 4 µM of gemcitabine for 48 h. (**D**) Representative images showed sphere formation (upper) and column graph presented number of spheres in the indicated PANC-1 cells (bottom). The total number of spheres was quantified across multiple replicate wells per condition, with the results shown as mean ± SEM. Scale bar: 20 μm. (**E**) The positive cells of CD133 expression and ALDH activity of the indicated PANC-1 cells were measured by flow cytometric analysis. (**F**) Kaplan-Meier plot of overall survival, relapse-free survival and recurrence-free survival in pancreatic cancer patients. Overall survival and relapse-free survival analyzed by the PROGgeneV2 online database. Recurrence-free survival in pancreatic cancer patients with gemcitabine treatment (*n* = 20), stratified by *LDHA* expression. The *LDHA* expression in patient samples was classified according to the individual ΔCP values of *LDHA* relative to *GAPDH*, where the lower ΔCP values indicate higher expression of *LDHA*. The median of individual ΔCP values of patient samples was used as cut-off values to define high and low expression. *P* = 0.0009 (Log-rank test). (**G**) There was a positive correlation between *LDHA* and CSC marker expression, including *CD44*, *ALDHA1* and *KLF4*, as determined by RT-qPCR in pancreatic cancer patients treated with gemcitabine (*n* = 20). The Pearson’s correlation coefficient, *r* and *P* values, are shown in each panel. The results are presented as the means ± s.e.m. of three independent experiments. *P < 0.05, **P < 0.01 and ***P < 0.001 (two-tailed Student’s *t-*test)

To investigate the underlying mechanisms involved in gemcitabine resistance in PDAC, we generated two gemcitabine-resistant PDAC cell lines, PANC-1/GEM and MIA PaCa-2/GEM (Supplementary Fig. 1A, B), and found that both had higher CD133 expression and ALDH activity, as well as *SOX2*, *KLF4* and *Nanog* CSC genes expression, compared with their parental PANC-1 and MIA PaCa-2 cells (Supplementary Fig. 1C, D). We further analyzed and compared the metabolites between PANC-1/GEM and PANC-1 cells by LC/MS and observed that PANC-1/GEM cells produced higher amount of lactic acid than PANC-1 cells (Supplementary Fig. 2A). We also found that PANC-1/GEM and MIA PaCa-2/GEM cells had higher lactate production (Supplementary Fig. 2B), LDH enzyme activity (Fig. 1A, left) and increased mRNA and protein expression of LDHA than their parental cells (Fig. 1A, right). In addition, we found that PANC-1 cells forming spheres in the sphere formation assay exhibited higher levels of LDH activity and LDHA expression, but not LDHB expression, compared with adherent PANC-1 cells. (Fig. 1B). Consistent results were shown in another PDAC cell line, SUIT-2 (Supplementary Fig. 3A), implying that gemcitabine-resistant and cancer stem-like PANC-1 cells have enhanced lactate production due to increased LDHA expression.

To validate whether LDHA is involved in the gemcitabine resistance of PDAC cells, we genetically modulated the LDHA expression in PANC-1, PANC-1/GEM, MIA PaCa-2 and MIA PaCa-2/GEM cells (Fig. 1C, upper). The results showed that overexpression of LDHA in PANC-1 and MIA PaCa-2 cells increased the gemcitabine resistance, and knocking down LDHA in gemcitabine-resistant cells significantly reduced their acquired resistance to gemcitabine (Fig. 1C, bottom), as well as in BxPC-3 and SUIT-2 cells (Supplementary Fig. 3B). We also found the sphere formation abilities (Fig. 1D), CSC phenotypes (Fig. 1E; Supplementary Fig. 3C) and tumor initiating capacity (Supplementary Table 1) were positively regulated by LDHA, suggesting that LDHA plays a critical role in the response to gemcitabine and the CSC properties of PDAC cells. By examining the Oncomine database, we observed that *LDHA* was significantly increased in human pancreatic cancer compared with normal pancreatic tissue (Supplementary Fig. 4), and positively correlated with high grade, advanced stage and metastatic pancreatic cancer (Supplementary Fig. 5). It also correlated with the expression of CSC markers, including *CD133*, *CD44*, *SOX2*, *OCT4*, *KLF4* and *Nanog* (Supplementary Table 2). Moreover, we analyze the LDHA expression correlates with pancreatic cancer patients’ survival rates by PROGgeneV2 online database and found that high expression levels of LDHA have lower overall survival and relapse-free survival in pancreatic cancer patients. We further used our clinical pancreatic cancer specimens to analyzed the *LDHA* levels in pancreatic cancer patients receiving treatment with gemcitabine and observed that patients with better overall-survival (OS) and recurrence-free survival (RFS) showed lower level of *LDHA* expression (Fig. 1F). Additionally, we found that the expression of *CD44*, *ALDHA1* and *KLF4* showed significant and positive correlations with the *LDHA* expression in this cohort (Fig. 1G). These results suggest that LDHA has a crucial role in promoting gemcitabine resistance and enhancing the CSC population of PDAC.

### miR-4259 suppresses LDHA expression

**Fig. 2:**
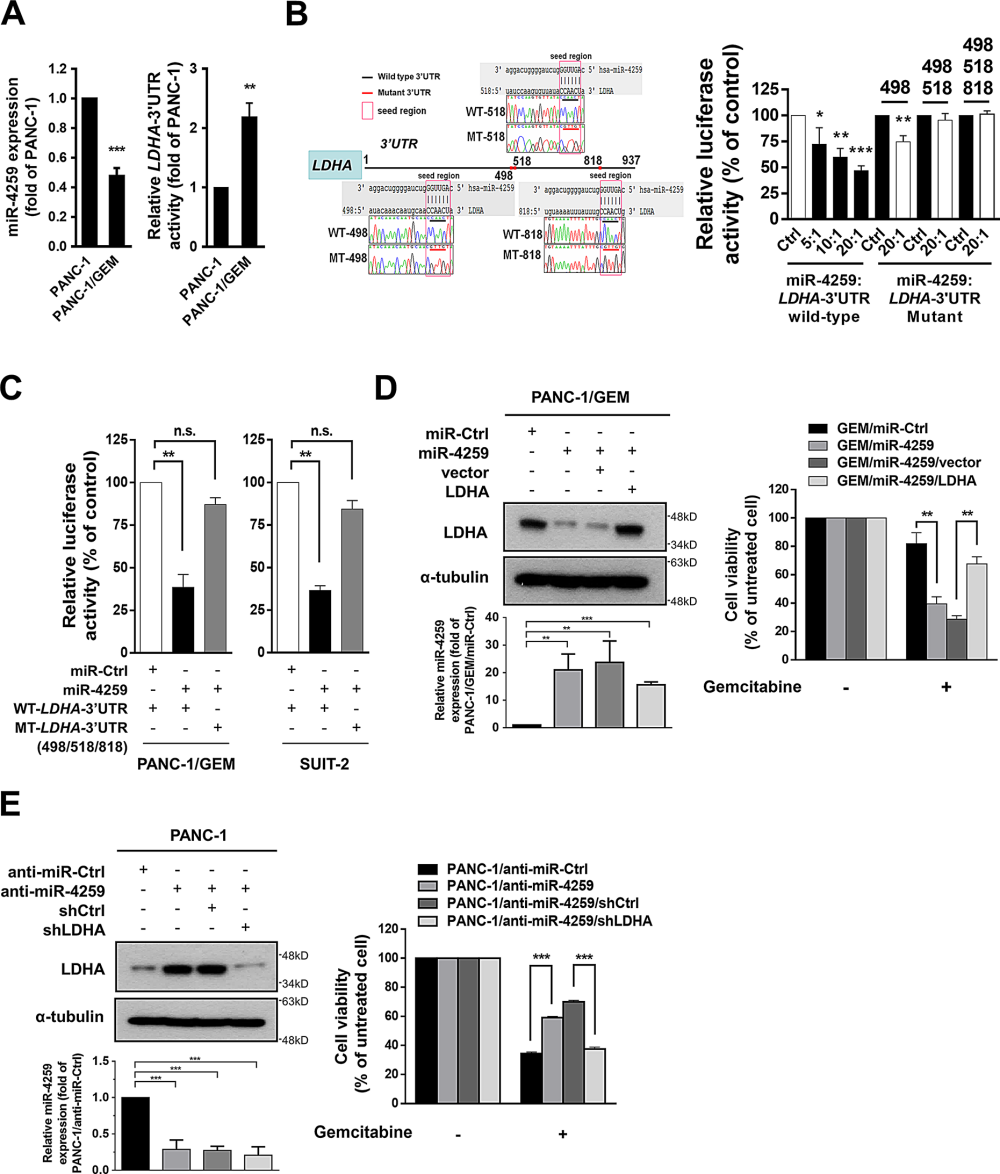
miR-4259 targets the *LDHA* 3’UTR and inhibits LDHA-mediated gemcitabine resistance in PDAC. (**A**) The miR-4259 expression (left) and *LDHA*-3’UTR luciferase activity (right) in PANC-1 and PANC-1/GEM cells was measured by RT-qPCR and a luciferase reporter assay, respectively. The RT-qPCR data were normalized to the level of *U47* RNA in each individual sample. (**B**) A schematic diagram representing the predicted miR-4259-binding sequences or the mutated versions of the miRNA (left). The luciferase reporter activity (right) of the *LDHA*-3’UTR wild-type (+ 1 ~ + 937) and *LDHA*-3’UTR mutant reporters (mutant sites: 498, 498/518 and 498/518/818) were measured by a dual-luciferase reporter assay in HEK-293T cells transfected with miR-4259 and a reporter at different ratios. (**C**) The luciferase reporter activity of the *LDHA*-3’UTR wild-type and *LDHA*-3’UTR mutant reporters (triple-mutant sites, 498/518/818) in PANC-1/GEM and SUIT-2 cells and their expression of miR-4259. (**D**) The LDHA and miR-4259 expression (left) of PANC-1/GEM cells transfected with the indicated plasmids were analyzed by Western blotting and RT-qPCR, respectively. The cell viability (right) of these transfectants in the presence of gemcitabine treatment was measured by the MTT assay. (**E**) The LDHA and miR-4259 expression (left) of PANC-1 cells were analyzed by Western blotting and RT-qPCR, respectively. The cell viability (*right*) of these transfectants in the presence of gemcitabine treatment was measured by the MTT assay. The results are presented as the means ± s.e.m. of three independent experiments. *P < 0.05, **P < 0.01, ***P < 0.001 and n.s. not significant (two-tailed Student’s *t*-test)

To identify underlying regulators involved in gemcitabine resistance and CSC properties, we compared the miRNA expression profiles between PANC-1/GEM and PANC-1 cells (Supplementary Fig. 6A) and predicted several candidate miRNAs which may target *LDHA* by multiple software programs, including TargetScan, miRanda, and DIANA-MICROT (Supplementary Fig. 6B). We used a combination of the miRNA microarray results and software predictions, and confirmed that PANC-1/GEM cells had lower expression of miR-4259 and a higher level of luciferase reporter activity at the wild-type 3’untranslated region (UTR) of *LDHA* mRNA (*LDHA*-3’UTR wild-type, + 1 ~ + 937 bp) than PANC-1 cells (Fig. 2A). This indicates that miR-4259 might be involved in the regulation of *LDHA* expression in PDAC cells.

To further examine whether miR-4259 directly targets to *LDHA*, we constructed a luciferase reporter vector harboring mismatches in the three predicted miR-4259-binding sites on 498, 518 and 818 (*LDHA* 3’-UTR mutants, MT-498, MT-518 and MT-818; Fig. 2B, left) and transfected these vectors into HEK293T cells at different miRNA-to-reporter ratios. Co-transfection of the pLmiR-4259 and WT-*LDHA*-3’UTR plasmids resulted in dose-dependent suppression of the luciferase activity (Fig. 2b, right). The mutant affecting the 498 site of the *LDHA*-3’UTR partially recovered the miR-4259-reduced luciferase activity, and the double-mutant of the 498/518 sites and triple-mutant (498/518/818 sites) of the *LDHA*-3’UTR had fully recovered the effects of miR-4259 (Fig. 2B, right). Gemcitabine-resistant PDAC cells showed higher luciferase activity of WT-*LDHA*-3’UTR than their parental cells, but not MT-*LDHA*-3’UTR (Supplementary Fig. 7A); overexpression of miR-4259 in PDAC cells also significantly reduced the luciferase activity of WT-*LDHA*-3’UTR, but not that of the MT-*LDHA*-3’UTR (Fig. 2C), showing that miR-4259 targets to the 3’UTR of *LDHA* and downregulates *LDHA* expression.

To determine the effects of miR-4259 on LDHA protein expression and gemcitabine resistance, we overexpressed miR-4259 in PANC-/GEM cells and found that the LDHA expression (Fig. 2D, left), LDH activity, lactate production (Supplementary Fig. 7B), tumor initiating capacity (Supplementary Table 3) and gemcitabine resistance (Fig. 2D, right; Supplementary Fig. 7D, E,F) were repressed by miR-4259. These effects were restored by expressing LDHA in PANC-1/GEM/miR-4259 cells (Fig. 2D; Supplementary Fig. 7B). Consistently, inhibition of miR-4259 by a specific antagomiR, anti-miR-4259, significantly increased the LDHA expression (Fig. 2E, left), LDH activity, lactate production (Supplementary Fig. 7C), and enhanced gemcitabine resistance (Fig. 2E, right). The anti-miR-4259-induced acquired gemcitabine resistance was abolished by further knockdown of LDHA (Fig. 2E). These results demonstrate that miR-4259 is crucial for regulating the LDHA expression and LDHA-mediated gemcitabine tolerance of pancreatic cancer cells.

### miR-4259 is transcriptionally regulated by FOXO3a

**Fig. 3:**
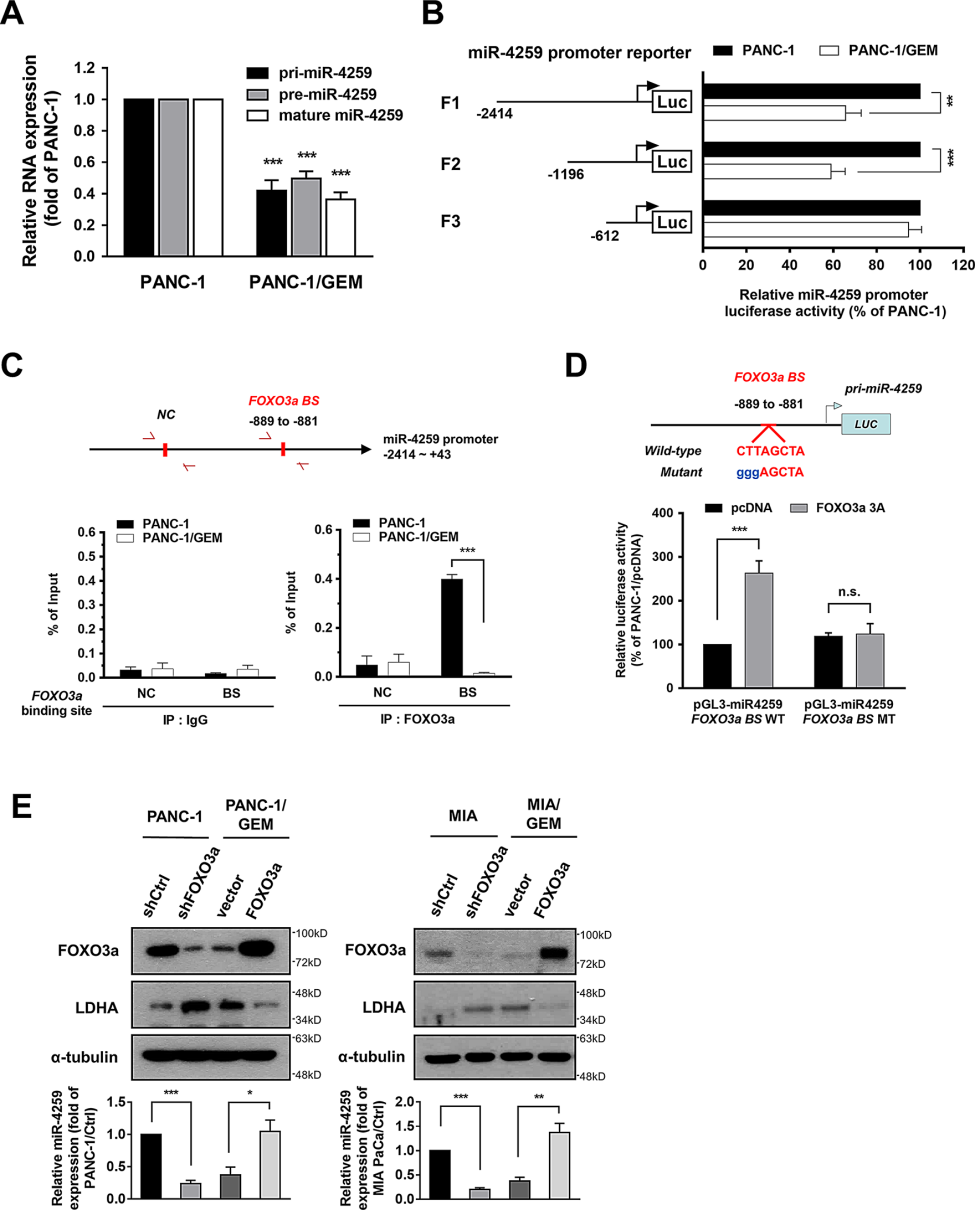
miR-4259 expression is transcriptionally regulated by FOXO3a. (**A**) The results of RT-qPCR analysis of pri-miR-4259, pre-miR-4259 and mature miR-4259 expression levels in PANC-1 and PANC-1/GEM cells. (**B**) The luciferase promoter reporter assays used a ~ 2.4-kb fragment upstream of the start codon of the miR-4259 gene (F1-F3). PANC-1 and PANC-1/GEM cells were transfected with the reporter constructs and the luciferase activity was measured after transfection. (**C**) Chromatin/protein lysates were extracted from PANC-1 and PANC-1/GEM cells, following immunoprecipitation with antibodies specific for FOXO3a, and determined by RT-qPCR analysis. FOXO3a binding site (BS); negative control (NC). (**D**) A schematic diagram showing the FOXO3a binding sequences or mutated versions of the miR-4259 promoter (upper). The luciferase activity was measured by a dual-luciferase reporter assay (bottom). FOXO3a 3 A, constitutively active FOXO3a. (**E**) The LDHA, FOXO3a (upper) and miR-4259 (bottom) expression in the paired PANC-1 cells (left panel) and paired MIA PaCa-2 cells (right panel) were analyzed by Western blotting and RT-qPCR, respectively. The results are presented as the means ± s.e.m. of three independent experiments. *P < 0.05, **P < 0.01,, ***P < 0.001 and n.s. not significant (two-tailed Student’s *t* test)

miRNA expression is controlled by a series of factors that regulate primary miRNA transcription, processing, maturation, and degradation. We found that primary, precursor and mature forms of miR-4259 were downregulated in PANC-1/GEM cells compared with PANC-1 cells (Fig. 3A), suggesting that the reduction of miR-4259 in PANC-1/GEM cells may occur through transcriptional regulation. We constructed serial miR-4259 promoter reporters (F1-F3) and found that the luciferase activities of the F1 and F2 miR-4259 promoter reporters, but not the F3 reporter, were significantly reduced in PANC-1/GEM cells compared with PANC-1 cells (Fig. 3B).

We subsequently analyzed the promoter region (-1196 ~ -612) of miR-4259 to search for response elements using the TESS 2.0/TFSEARCH software. We found an interesting element, a FOXO3a binding site, in this region (Fig. 3C, upper). The FOXO3a transcription factor has been shown to regulate miRNAs expression in adrenocortical and breast cancer cells [20]. We verified the interaction between FOXO3a and the miR-4259 promoter by a chromatin immunoprecipitation assay. We found that there was less FOXO3a binding to the miR-4259 promoter on the FOXO3a binding site (*FOXO3a BS*) in PANC-1/GEM cells than in PANC-1 cells (Fig. 3C, bottom).

We then investigated whether FOXO3a is involved in regulating miR-4259 promoter activity by the miR-4259 promoter-reporter with the mutated FOXO3a binding site (Fig. 3D, upper). Compared with PANC-1 cells transfected with the vector alone, the luciferase assay demonstrated that the constitutively active FOXO3a (FOXO3a (3 A), the three AKT phosphorylation sites on FOXO3a were mutated to alanines) stimulated the miR-4259 wild-type promoter activity, but not the activity of the miR-4259 FOXO3a binding site mutant (*FOXO3a BS* MT) promoter (Fig. 3D, bottom). To define the role of FOXO3a in miR-4259 regulation and LDHA expression, we knocked down FOXO3a in PANC-1 and MIA PaCa-2 cells, and found that shFOXO3a reduced miR-4259 expression and increased LDHA expression (Fig. 3E). In addition, overexpression of FOXO3a in PANC-1/GEM and MIA PaCa-2/GEM cells increased miR-4259 expression and suppressed LDHA expression (Fig. 3E). We found that nuclear FOXO3a expression was decreased in PANC-1/GEM cells compared with PANC-1 cells and FOXO3a expression positively correlated with PTEN level and inversely correlated with phosphor-Akt and LDHA expression in PANC-1 and PANC-1/GEM cells (Supplementary Fig. 8A, B), suggesting that increased FOXO3a expression and nuclear localization in PANC-1 cells might result from PTEN and PI3K/AKT regulation. Additionally, expression of myr-AKT in PANC-1 cell decreased FOXO3a and miR-4259 expression, increased LDHA expression and cell viability; further inhibition of AKT activity by AKT inhibitor (AKTi) reversed the effects of myr-AKT in PANC-1/myr-AKT cells (Supplementary Fig. 8C). Treatment of AKTi in PANC-1/GEM cells increased FOXO3a and miR-4259 expression, reduced LDHA expression and decreased cell viability, and further expression of miR-4529 antagomiR (anti-miR-4259) restored LDHA expression and cell viability of PANC-1/GEM cells with AKTi treatment (Supplementary Fig. 8D). Taken together, these results indicated that miR-4259 is transcriptionally regulated by FOXO3a to further suppress the LDHA expression in pancreatic cancer cells.

### FOXO3a/miR-4259 regulate LDHA-induced gemcitabine resistance

**Fig. 4:**
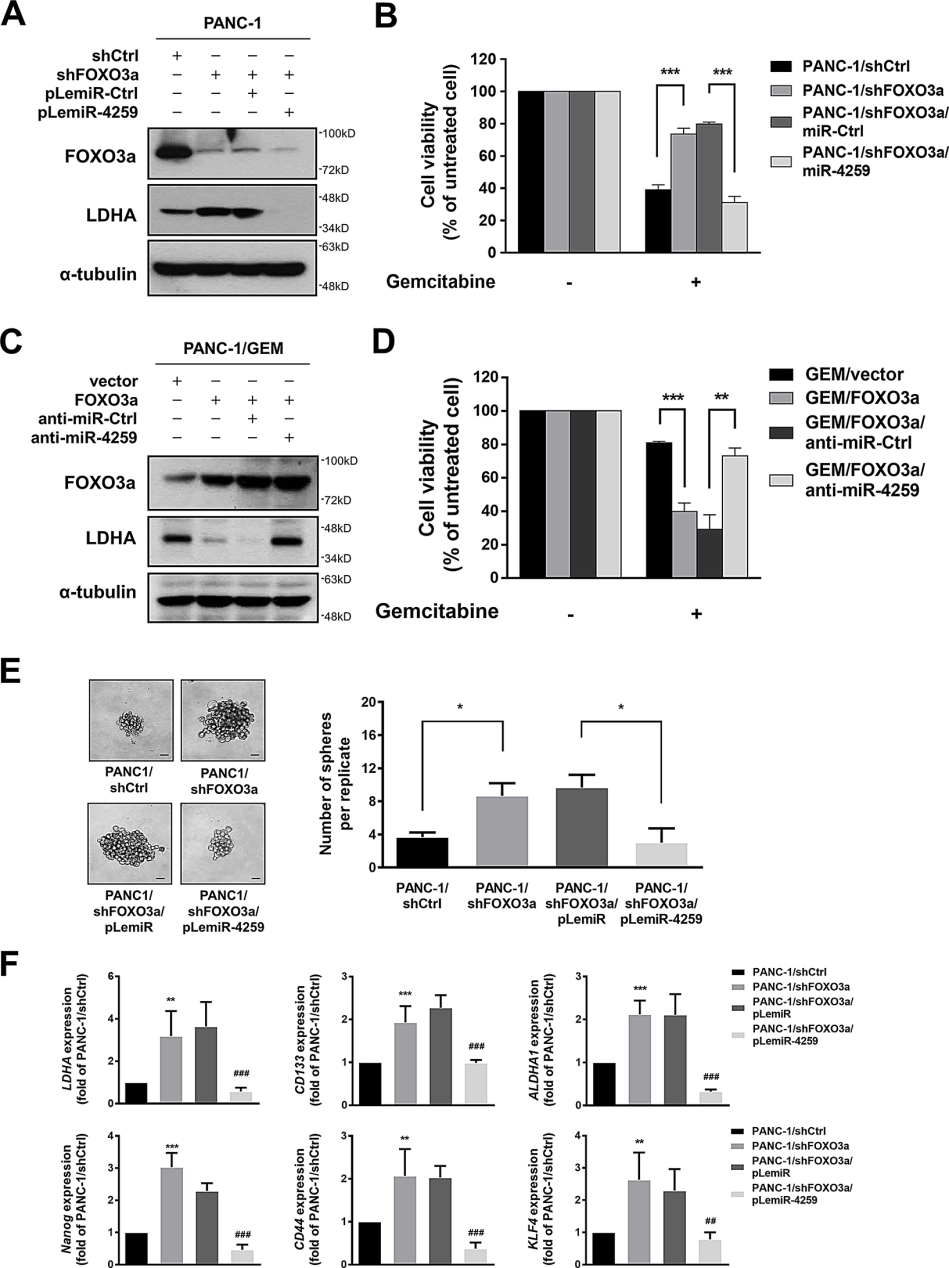
FOXO3a/miR-4259 regulates LDHA-mediated gemcitabine resistance and cancer stemness. (**A**) The LDHA and FOXO3a expression of the indicated PANC-1 cell lines was analyzed by Western blotting, and (**B**) The cell viability of these transfectants treated with gemcitabine was measured by the MTT assay. α-tubulin was used as the internal protein loading control. (**C**) The LDHA and FOXO3a expression of the indicated cell lines was analyzed by Western blotting, and (**D**) The cell viability of these transfectants treated with gemcitabine was measured by the MTT assay. α-tubulin was used as the internal protein loading control. (**E**) Sphere formation (left) and the number of spheres (right) for the indicated PANC-1 cell lines. The total number of spheres was quantified across multiple replicate wells per condition, with the results shown as mean ± SEM. Scale bar: 20 μm. (**F**) The *LDHA* and CSC marker expression (*CD133*, *CD44*, *ALDHA1*, *Nanog* and *KLF4*) were measured by RT-qPCR in the indicated PANC-1 cell lines. The results are presented as the means ± s.e.m. of three independent experiments. *P < 0.05, **P < 0.01 and ***P < 0.001 (two-tailed Student’s *t* test)

Next, we studied the role of FOXO3a in the gemcitabine sensitivity and CSC phenotypes in PDAC cells. Knocking down FOXO3a in PANC-1 cells increased the LDHA protein expression (Fig. 4A), LDH activity, lactate production (Supplementary Fig. 9a) and contributed to increased gemcitabine tolerance (Fig. 4B). Increasing the miR-4259 level to reduce LDHA expression counteracted the chemoresistance of the PANC-1/shFOXO3a cells to gemcitabine (Fig. 4A, B). On the other hand, enforced expression of FOXO3a in PANC-1/GEM cells decreased the LDHA expression (Fig. 4C), CSC phenotypes (Supplementary Fig. 9B), tumour initiating capacity (Supplementary Table 4) and suppressed gemcitabine resistance (Fig. 4d and Supplementary Fig. 9C, D,E); further abolishment of miR-4259 by anti-miR-4259 significantly restored the effects of FOXO3a in PANC-1/GEM cells (Fig. 4C, D; Supplementary Fig. 9B and Supplementary Table 4). These results suggest that FOXO3a-regulated miR-4259 expression directly reduces the LDHA level and decreases the gemcitabine resistance in PDAC cells. In addition, knocking down FOXO3a led to increased sphere formation (Fig. 4E) in PANC-1 cells. Stable expression of miR-4259 suppressed the shFOXO3a-induced sphere formation (Fig. 4E), *LDHA* expression (Fig. 4F) and expression of CSC genes, including *CD133*, *CD44*, *Nanog*, *KLF4* and *ALDHA1* (Fig. 4F), suggesting that the FOXO3a/miR-4259 axis regulates LDHA-induced gemcitabine resistance and the stemness of PDAC cells.

**Fig. 5:**
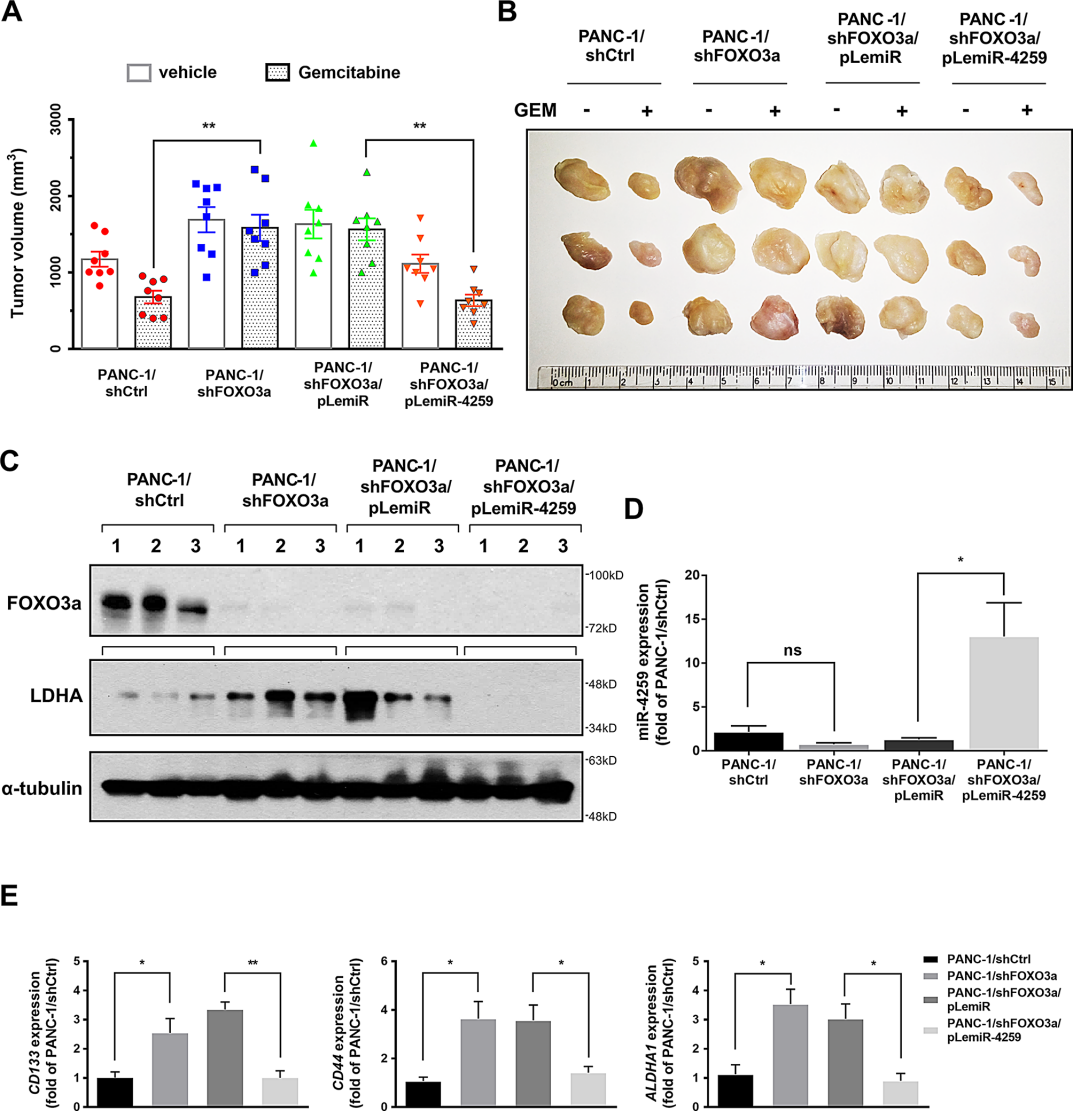
FOXO3a/miR-4259 reduces the LDHA expression and enhances the gemcitabine sensitivity of PDAC in vivo. (**A**) Mice were subcutaneously implanted with the (PANC-1/shCtrl, PANC-1/shFOXO3a, PANC-1/shFOXO3a/pLemiR, PANC-1/shFOXO3a/pLemiR-4259) until the resulting tumours reached approximately 200 mm^3^. The mice were then intraperitoneally treated with vehicle or 50 mg/kg gemcitabine once a week. Each column represents the means ± s.e.m. of the tumour volumes of eight mice in each group. The tumour volume was calculated as described in the Methods section. (**B**) The indicated tumours from mice treated with the vehicle and gemcitabine for twenty-four days were dissected from the surrounding tissue. (**C**) Western blotting was performed to confirm the expression of FOXO3a and LDHA in the indicated groups of tumour samples. α-tubulin was used as a loading control. The relative expression of (**D**) miR-4259, (**E**) *CD133*,* CD44* and *ALDHA1* were measured by RT-qPCR analysis. The results are shown as the means ± s.e.m. of three independent experiments. *P < 0.05, ** *P* < 0.01 and n.s. not significant (two-tailed Student’s *t* test)

We validated these findings in an in vivo model, and observed that knocking down FOXO3a significantly enhanced the chemoresistance of the xenograft tumours to gemcitabine (Fig. 5A, B). Enforced expression of miR-4259 in PANC-1/shFOXO3a cells re-sensitized the xenograft tumours to gemcitabine in vivo. These tumours were subsequently examined for their expression of FOXO3a, LDHA and miR-4259 by Western blotting (Fig. 5C) and RT-qPCR (Fig. 5D), respectively. Together, these results indicated that LDHA expression was regulated by FOXO3a/miR4259 signaling and positively correlated with gemcitabine tolerance in PDAC tumours. Additionally, we analyzed the expression of CSC genes in these tumours and found that the expression levels of *CD133*, *CD44* and *ALDHA1* were consistent with the effects of FOXO3a/miR4259 signaling on LDHA expression (Fig. 5E). Taken together, the in vitro and in vivo experimental results revealed that LDHA expression and LDHA-mediated CSC phenotypes were suppressed by FOXO3a/miR-4259 signaling and contributed to enhancing the gemcitabine sensitivity of PDAC.

### Clinical significance of FOXO3a and miR-4259 expression

**Fig. 6:**
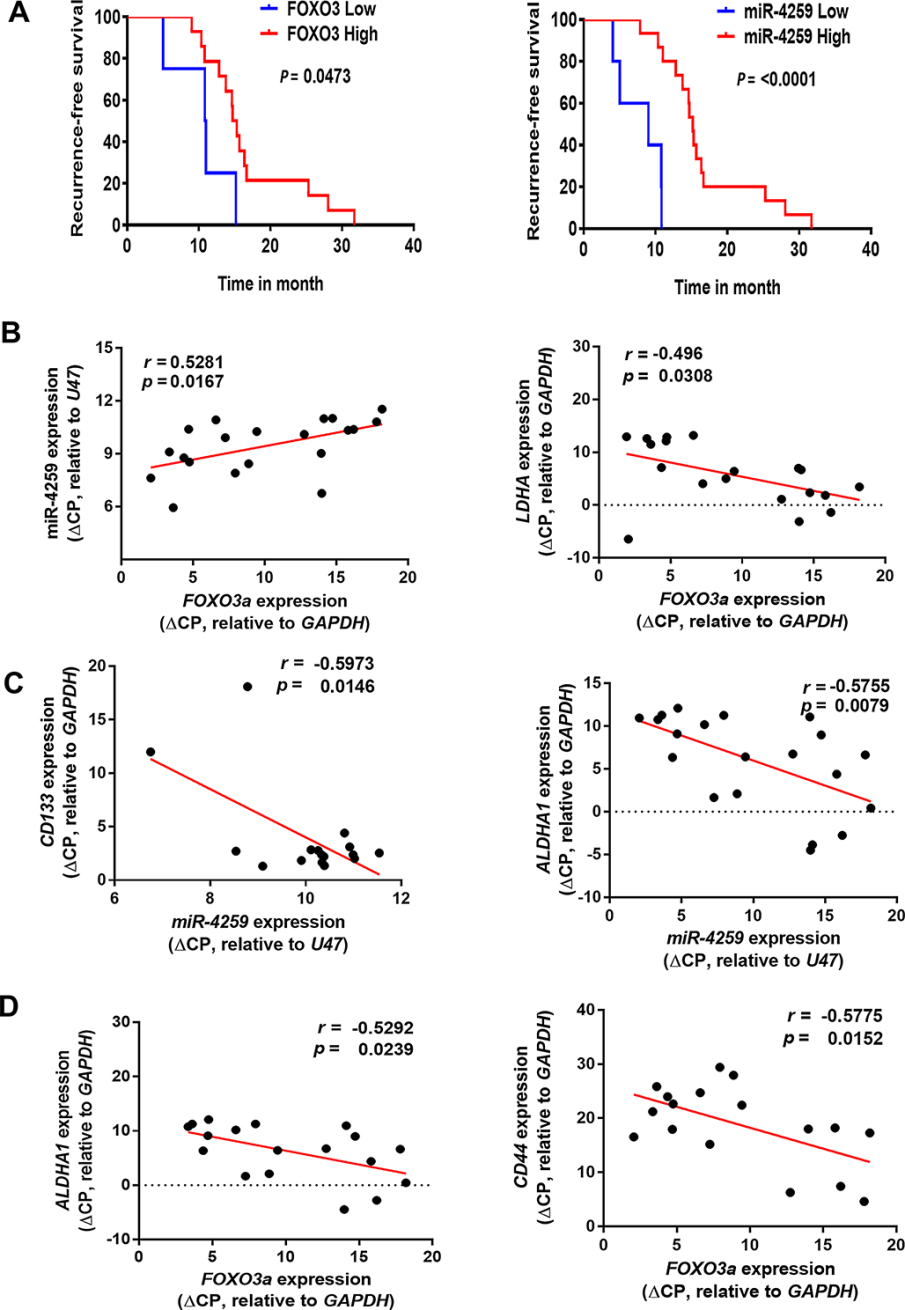
The clinical significance of *FOXO3a* and miR-4259 expression in pancreatic cancer. (**A**) Kaplan-Meier plot of recurrence-free survival in pancreatic cancer patients (*n* = 20), stratified by miR-4259 (left) and *FOXO3a* (right). The *FOXO3a* and miR-4259 expression in the patient samples was classified according to the individual ΔCP values of *FOXO3a* and miR-4259 relative to those of *GAPDH* and *U47*, respectively, where a lower ΔCP value indicated higher expression of *FOXO3a* or miR-4259. The median of individual ΔCP values of patient samples was used as cut-off values to define high and low expression. (**B**) *FOXO3a* positively correlated with miR-4259 (left), and inversely correlated with *LDHA* (right) in pancreatic cancer samples from patients treated with gemcitabine. There was an inverse correlation between CSC marker expression and miR-4259 (**C**), as well as with *FOXO3a* (**d**) in samples from pancreatic cancer patients. The Pearson’s correlation coefficient, *r* and *P* values, are shown in each panel

To elucidate the clinical significance of FOXO3a and miR-4259 expression in the sensitivity pancreatic cancer to gemcitabine, we respectively analyzed the expression of FOXO3a and miR-4259 in pancreatic cancer patients who has been treated with gemcitabine-based adjuvant therapy and found that patients with high level of FOXO3a or miR-4259 expressions have better RFS outcomes than those low expression of FOXO3a or miR-4259 in tumours (Fig. 6A). In addition, the expression of *FOXO3a* positively correlated with the miR-4259 expression (Fig. 6B, left), and inversely correlated with the *LDHA* expression (Fig. 6B, right) in this cohort.

Moreover, we performed another Oncomine database analysis and found that *FOXO3a* expression inversely correlated with *LDHA* expression (Supplementary Table 5) and negatively correlated with the expression of CSC-related pluripotency genes, including *CD133*, *CD44*, *SOX2*, *OCT4*, *KLF4* and *Nanog* (Supplementary Table 6). Consistently, we observed that the expression levels of *CD133* and *ALDHA1* were inversely correlated with miR-4259 (Fig. 6C) expression, and the levels of, *ALDHA1* and *CD44* were significantly and negatively corrected with *FOXO3a* (Fig. 6D) expression in our PDAC cohort. These clinical observations suggest that the expression of FOXO3a and miR-4259 positively correlates with recurrence-free survival outcomes in pancreatic cancer patients treated with gemcitabine and inversely correlates with the expression of LDHA and CSC markers within this cohort.

## Discussion

**Fig. 7:**
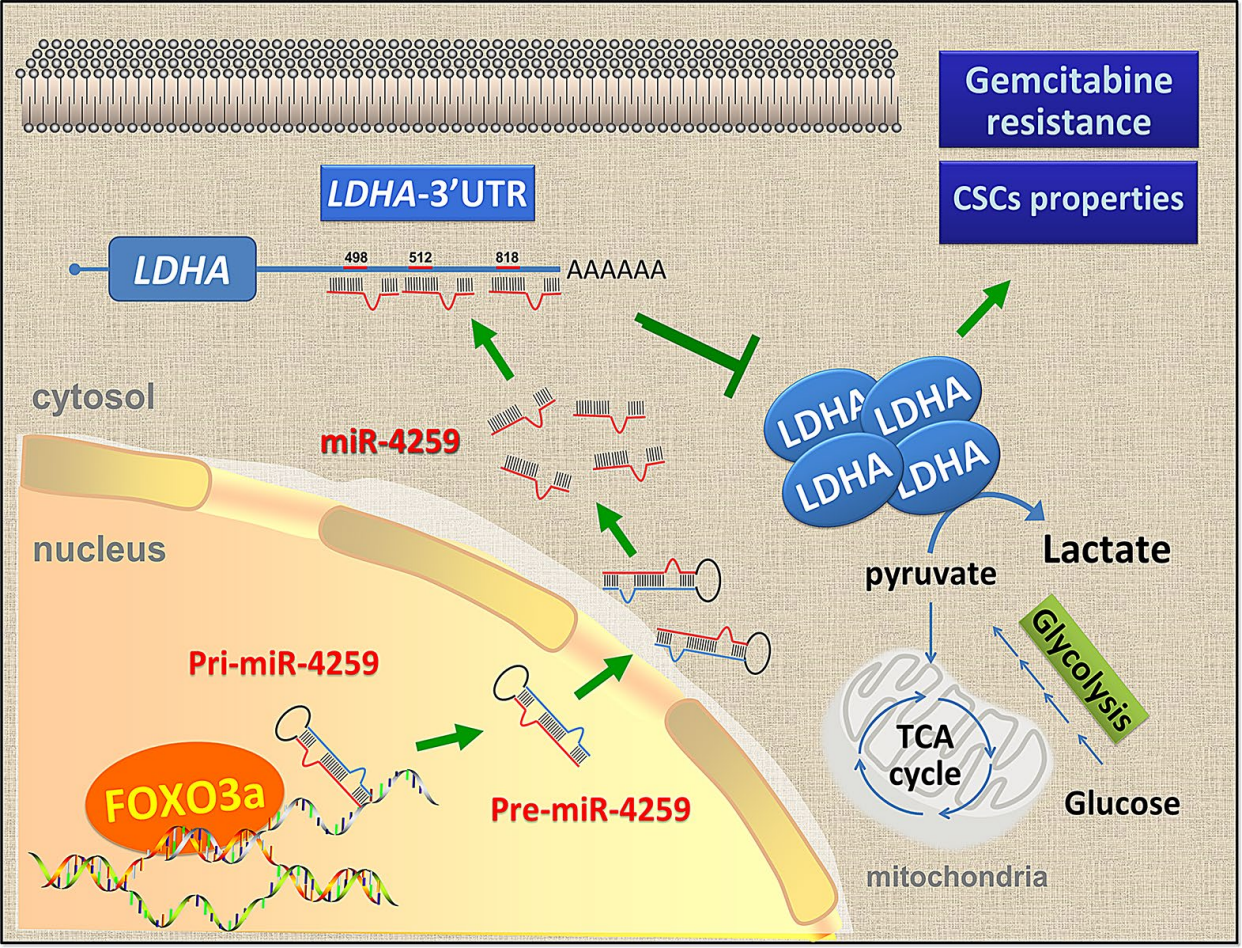
A schematic model illustrating the suppression of LDHA by FOXO3a through the transcriptional induction of miR-4259 in pancreatic cancer, and how this affects stemness and gemcitabine resistance

Accumulating studies have revealed that cancers become advanced and resistant to chemotherapy or radiotherapy due to their altered metabolism [21]. LDH is essential to maintain a high glycolysis rate in the early stage of glycolysis [22] and LDH is a key enzyme required for oxidative metabolism shift to glycolytic metabolism. Targeting LDHA might serve as an effective and attractive strategy for cancer treatment. In this study, we uncovered that the repression of LDHA by FOXO3a-mediated miR-4259 expression suppresses the gemcitabine resistance and cancer stemness of pancreatic cancer (Fig. 7). Our findings indicate that high levels of LDHA expression are correlated with poor recurrence-free survival in patients who received gemcitabine treatment; however, comparisons with patients not treated with gemcitabine were not included in this study.

Cancer cells express high level of LDHA to increase the rate of aerobic glycolysis, lactate and ATP production for rapid cell proliferation, which benefits cancer cells by avoiding the generation of oxidative stress. Previous studies found that reduction of lactate levels correlates with tumour growth suppression in animals with chemotherapy, such as 5-fluorouracil, cyclophosphamide, or combined PI3K/mTOR inhibitor and temozolomide treatment [23, 24], implying that reduced lactate levels after therapy may result from decreased glycolysis and cell death. The high lactate level is considered to increase extracellular acidity and acidic microenvironment induces ERK1/2 and p38 signaling and increases NF-κB activity [25], both signalings are crucial for increasing CD133 expression and CSC populations [26]. Acidic stress has been reported to induce the HIF protein expression and promote glioma CSC phenotypes, including *CD133*, *Nanog* and *Oct4* expression and sphere-forming ability [27]. Of note, lactate also induces NF-κB/IL-8 pathway activation [28] and stabilizes HIF-1α [29, 30], which might induce CD133 expression and promote CSC populations [31]. Taken together, LDHA may increase lactate production to activate the signaling pathways which critical for cancer stemness, such as NF-κB, IL-8 and HIF-1α, and further regulate the CSC properties. Recently, it has been reported that treatment of lactate in hepatocellular carcinoma cells promotes CD133 expression, induces *Nanog*, *Oct4* and *SOX2* genes expression and increases sorafenib resistance [32], and targeting LDHA by siRNAs markedly decreases spheroid formation capacity and reduces the expression of CSC genes and mesenchymal markers in cancers [32, 33], suggesting that LDHA facilitates aerobic glycolysis and might produce lactate to alter CSC characteristics and promote tumour progression.

Aberrant LDHA expression is commonly observed in cancers, and upregulation of LDHA plays an important role in tumorigenesis and malignant progression [7, 34]. LDHA expression is known to be transactivated by HIF-1 [35, 36], and elevated LDHA expression is positively associated with HIF-1 expression and worse survival outcomes in cancer patients [37, 38]. In addition, c-Myc binds to the *LDHA* promoter and upregulates *LDHA* expression to increase c-Myc-mediated transformation [39, 40]. Recent studies also find that FOXM1 upregulates LDHA transcription to enhance lactate production and promote cancer growth and metastasis [8], whereas KLF4 negatively regulates *LDHA* gene expression and is involved in the progression of pancreatic cancer [41]. Post-translational modification is crucial for LDHA regulation. For example, lysine acetylation of LDHA was found to stabilize the LDHA protein level and is also involved in the progression of pancreatic cancer [7, 42]. Other factors including lactate, cAMP, estrogen, ErbB2 and HSF-1 have been reported to influence LDHA expression and glycolysis in cancers [34], indicating that LDHA regulation is subtly controlled by a series of factors that need to be further investigated. Our present findings showed a mechanism underlying the regulation of *LDHA* expression, which involved the post-transcriptional regulation by miR-4259 in pancreatic cancer cells. We showed that this mechanism appears to be clinically significant in pancreatic cancer patients, where it was associated with the response to gemcitabine treatment and CSC marker expression. This suggests that the regulation of LDHA by microRNAs impacts the chemosensitivity and cancer stemness of pancreatic cancer.

Growing evidence has revealed that the levels of aberrant miRNAs are frequently altered in cancers [43], and several specific miRNAs, such as miR-21, miR-375, miR-143, miR-14 and miR-29b, have been demonstrated to directly target to genes encoding metabolic enzymes to control cancer metabolism [11]. In a previous study, the expression of lactate dehydrogenase B (LDHB) was directly regulated by miR-375, which suppressed the proliferation and invasion of maxillary sinus squamous cell carcinoma [44]. Recently, direct targeting of LDHA by miR-34a was shown to re-sensitize colon cancer cells to 5-fluorouracil [45], and miR-34a was found to be silenced by the aberrant CpG methylation of its promoter in various cancers [46]. These studies suggest that the regulation of LDHA by miRNAs is important and contributes to various types of chemoresistance. Of note, we found that miR-4259 targeted to three conserved binding sites on the *LDHA*-3’UTR, and we further observed that the miR-4259 promoter region contained FOXO3a binding elements and was positively regulated by FOXO3a, implying that inhibiting FOXO3a/miR-4259 increases the LDHA protein level and its activity, leading to resistance to gemcitabine in PDAC.

The four human FOXO proteins, including FOXO1, FOXO3a, FOXO4 and FOXO6, contain winged-helix structures on their DNA binding domain and belong to a subgroup of the larger family of Forkhead-box-containing transcription factors [47]. FOXO3a is a tumour suppressor could be regulated by several crucial pathways, including PTEN/PI3K/Akt [19, 48–50], ERK/MAPK [51], and IKK [52] signaling pathway and modulation of FOXO3a has been reported to involve in the regulation of CSC properties, chemosensitivity and tumour initiation in cancers [16, 19, 53]. Above studies suggest that targeting PI3K/Akt/FOXO3a might suppress stem-like and chemotherapy-resistant cancer cells in the tumour and offer potential therapeutic strategies for poor treatment response of cancer patients. FOXO3a activation reduces HIF-1α accumulation under hypoxic conditions and decreases c-Myc stability [54], which affects the cell cycle, glycolysis and response to oxidative stress [55]. In addition, recent studies have indicated that both HIF-1α and c-Myc are able to bind to and transactivate the *LDHA* promoter, leading to the induction of LDH activity and lactate production [7]. Consistently, we found that FOXO3a drives miR-4259 expression to target *LDHA*-3’UTR and decreases LDHA expression and LDH activity in pancreatic cancer, suggesting that the regulation of LDHA is a finely tuned process that helps to balance the entry of pyruvate into aerobic glycolysis. FOXO3a is known to be a transcriptional factor responsible for miR-34b/c expression, which binds to the 3’UTR of *c-Myc* and disrupts colorectal tumorigenesis [56]. In addition, FOXO3a negatively regulates the expression of miR-21, which targets the 3’UTR of the Fas ligand (*FasL*), a pro-apoptotic factor, and enhances doxorubicin-induced apoptosis in lung cancer cells [57]. Moreover, FOXO3a transactivates miR-484 expression, which might downregulate cytidine deaminase, resulting in enhanced sensitivity of breast cancer to gemcitabine [58, 59]. These studies and our present findings suggest that FOXO3a might act not only as a tumour suppressor, but may also play an important role in regulating miRNAs involved in the metabolic and epigenetic reprogramming of cancers.

Our study specifically analyzed correlations between FOXO3a, miR-4259, LDHA, and CSC markers in pancreatic cancer patients who received gemcitabine treatment. However, a direct comparison with a no-gemcitabine control group was not performed, which limits the generalizability of our findings to untreated conditions. Future studies including such controls will provide more comprehensive insights into the regulatory relationships in the absence of chemotherapy.

## Conclusions

In summary, we herein demonstrated that miR-4259 directly targets to *LDHA*-3’UTR and reduces LDHA expression, leading to suppressed LDHA-mediated gemcitabine resistance and CSC properties. We also found the transcriptional regulation of miR-4259 by FOXO3a and the clinically relevant relationship among *LDHA*, miR-4259 and *FOXO3a* in pancreatic cancer that was related to the response to gemcitabine treatment. Our findings provide evidence of a novel function and regulatory mechanism of LDHA that is involved in the cancer stemness and gemcitabine resistance of pancreatic cancer. Our findings suggest that targeting the FOXO3a/miR-4259/LDHA pathway may serve as a new treatment for pancreatic cancer, especially in patients with a poor response to gemcitabine chemotherapy.

## Electronic supplementary material

Below is the link to the electronic supplementary material.

Supplementary Material 1

Supplementary Material 2

Supplementary Material 3

## Data Availability

No datasets were generated or analysed during the current study.

## Abbreviations

CSC: cancer stem cell
FOXO3a: Forkhead box O3a
GEM: gemcitabine
LC-MS: liquid chromatography-mass spectrometry
LDH: lactate dehydrogenase
LDHA: lactate dehydrogenase A
mRNA: message RNA
miRNA: microRNA
PCR: polymerase chain reaction
PDAC: pancreatic ductal adenocarcinoma
pri-miR-4259: primary miR-4259
pre-miR-4259: precursor miR-4259
RT-qPCR: reverse transcription-quantitative polymerase chain reaction
shRNA: short hairpin RNA
TCGA: The Cancer Genome Atlas
UTR: untranslated region
