# Supplementary material for: FOXO3a/miR-4259-driven LDHA expression as a key mechanism of gemcitabine sensitivity in pancreatic ductal adenocarcinoma
Source: Cancer Metab. 2025 Feb 10;13:7. doi: 10.1186/s40170-025-00377-3 (PMC11809001; doi:10.1186/s40170-025-00377-3)

Supplementary Figure 1

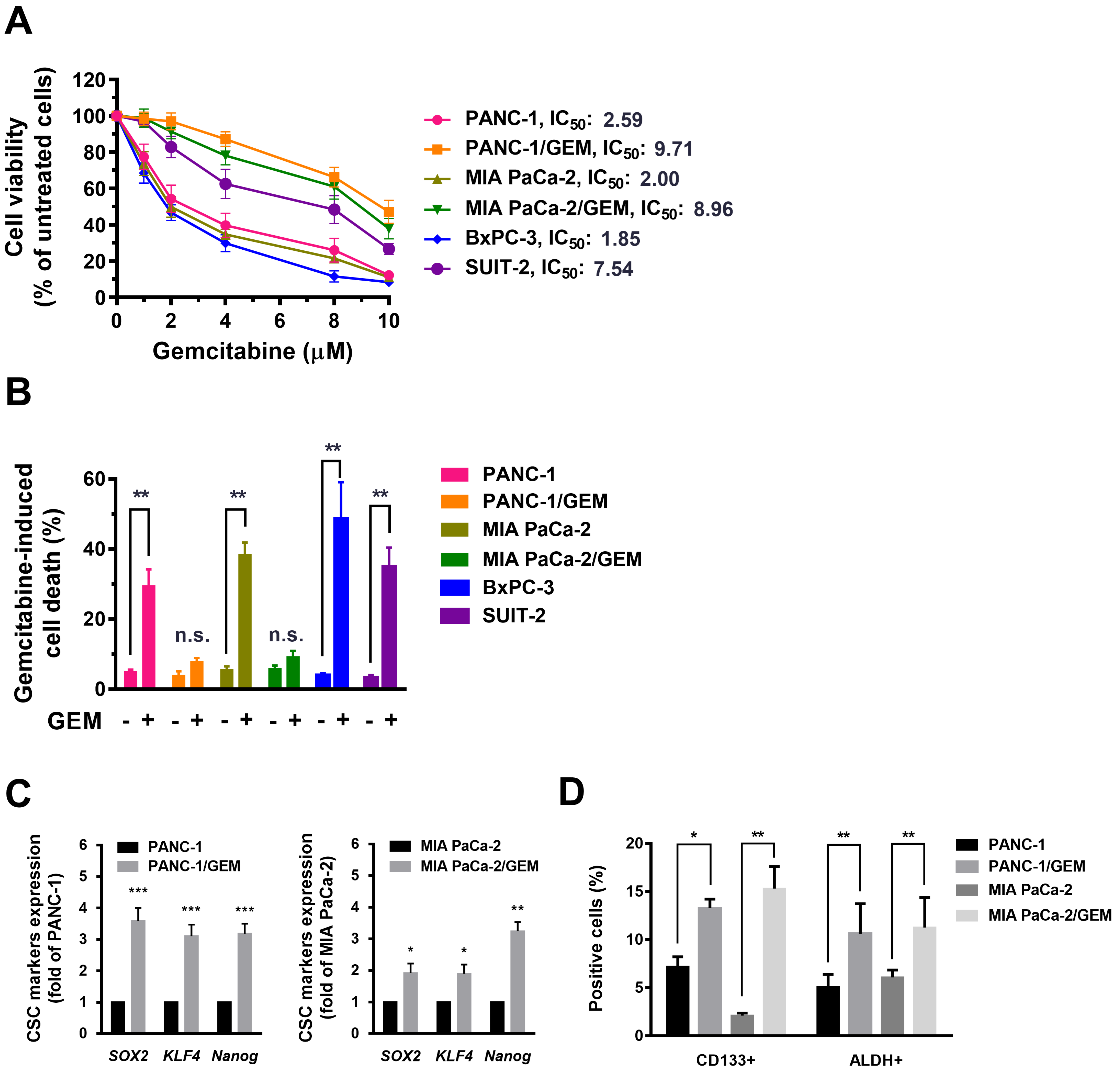

Supplementary Figure 2

A

Metabolites analysis of PANC-1/GEM compared with PANC-1 cells by LC/MS

	m/z	Database	Formula	ID	Fold by Change	*P* value
1	89.026	Biomolecules(Local)	C3H6O3	(1) L-Lactic acid	7.8	0.026
2	114.0888	Biomolecules(Local)	C6H11NO	(1) N-Acetylglutamine	1.5	0.043
3	116.0690	Biomolecules(Local)	C5H9NO2	L-Proline	6.6	0.022
4	118.0616	Biomolecules(Local)	C5H11NO2	(1) Trimethylglycine	1.8	0.027
5	133.0961	Biomolecules(Local)	C5H12N2O2	(1) Ornithine	5.6	0.040
6	147.0420	Biomolecules(Local)	C5H6O5	(1) alpha-Ketoglutaric acid	1.5	0.044
7	148.0540	Biomolecules(Local)	C5H9NO4	(1) L-Glutamic acid	1.5	0.026
8	160.0375	HMDB(online)*	C10H9NO	(1) Indoleacetaldehyde	1.5	0.029
9	158.9559	HMDB(online)	C5H6N2O4	(1) L-Dihydroorotic acid	2.1	0.015
10	226.9398	Phosphatidic Acids (Local)	C5H7O8P	(1) 1,2-diacyl-sn-glycero-	2.3	0.029
11	234.1006	Biomolecules(Local)	C10H18N2O5	(1) L-beta-aspartyl-L-leucine	4.9	0.010
12	307.0882	HMDB(online)	C9H14N2O7	(1) L-beta-aspartyl-L-glutamic acid	7.5	0.046
13	308.1605	HMDB(online)	C19H21N3O	(1) L-Arginosuccinic acid	5.2	0.029
14	325.1058	Biomolecules(Local)	C9H13N2O9P	(1) Uridine 5'-monophosph	1.5	0.037
15	335.1027	HMDB(online)	C10H14N2O6	(1) Ribothymidine	3.0	0.043
16	353.0694	HMDB(online)	C6H8O6	(1) D-Glucurono-6,3-lactone	1.6	0.021
17	425.1287	HMDB(online)	C19H24N2O9	(1) 6-Hydroxymelatonin glucuronide	1.7	0.045
18	455.1176	Biomolecules(Local)	C13H19N4O12P	(1) SAICAR	5.5	0.007
19	515.1444	HMDB(online)	C9H14N2O6	(1) L-alpha-Aspartyl-L-hydroxyproline	1.4	0.026
20	600.2783	HMDB(online)	C83H149N3O34	(1) Ganglioside GD2	1.6	0.009

*HMDB(online), Human Metabolome Database (Version 3.6)

B

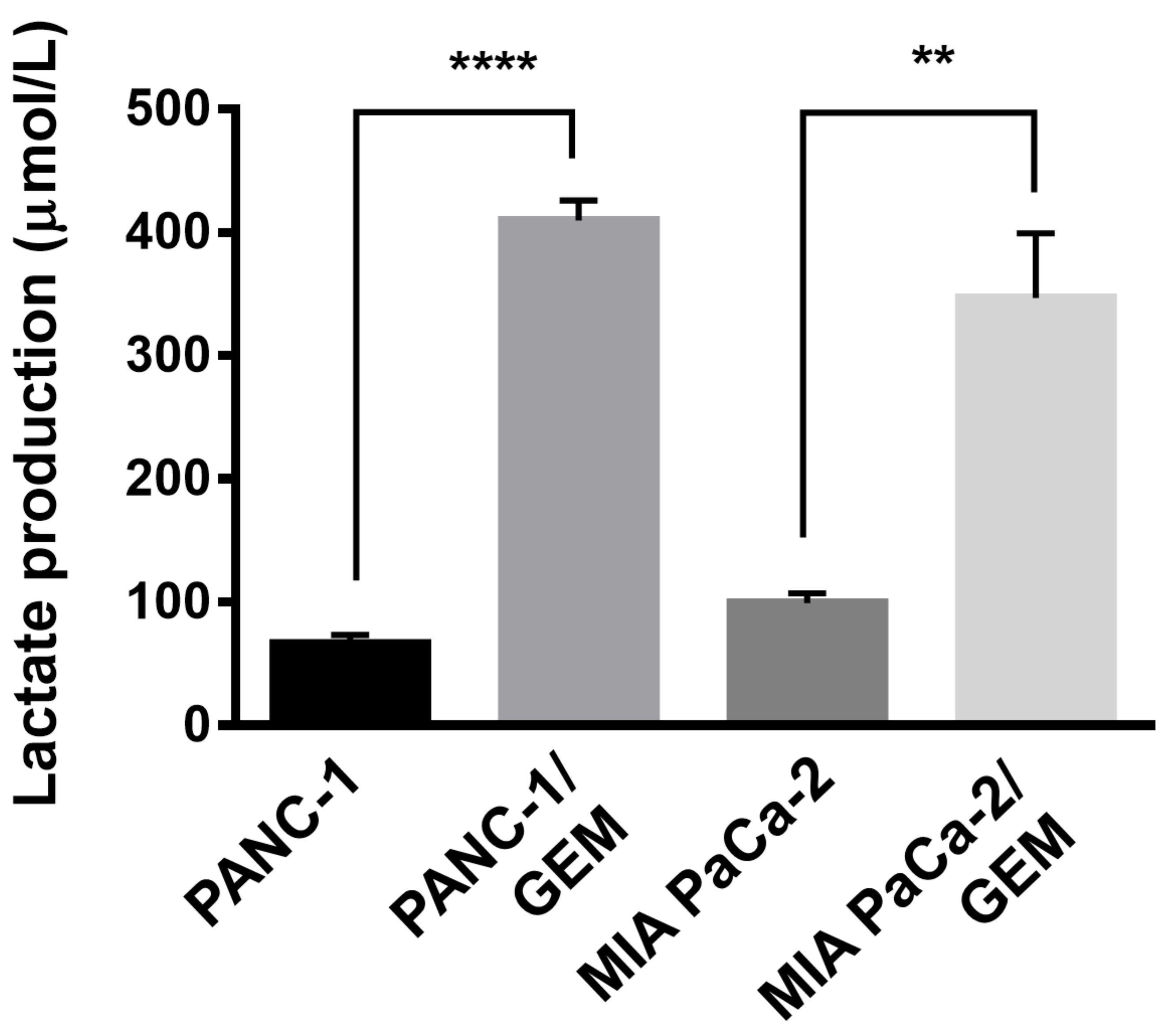

Supplementary Figure 3

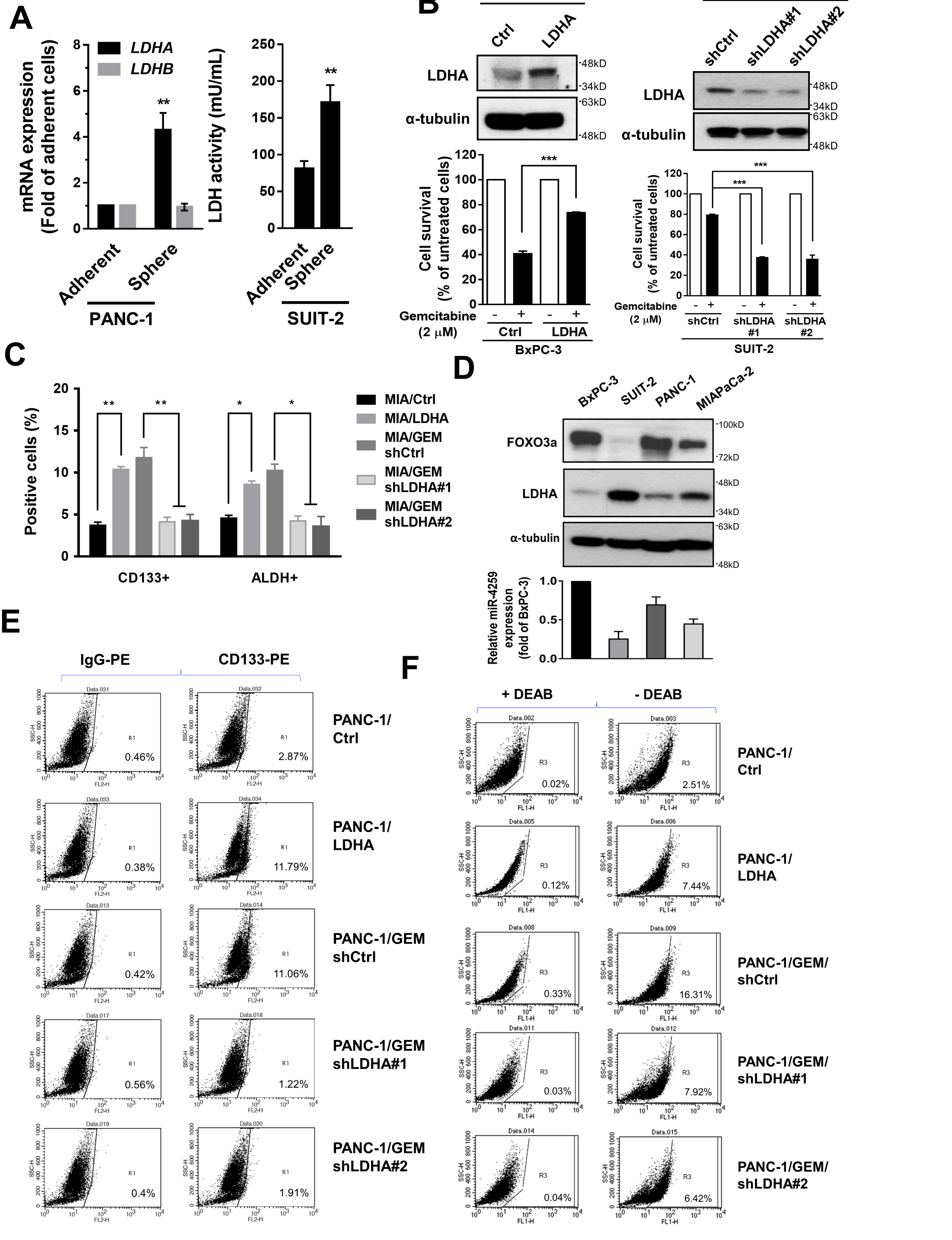

Supplementary Figure 4

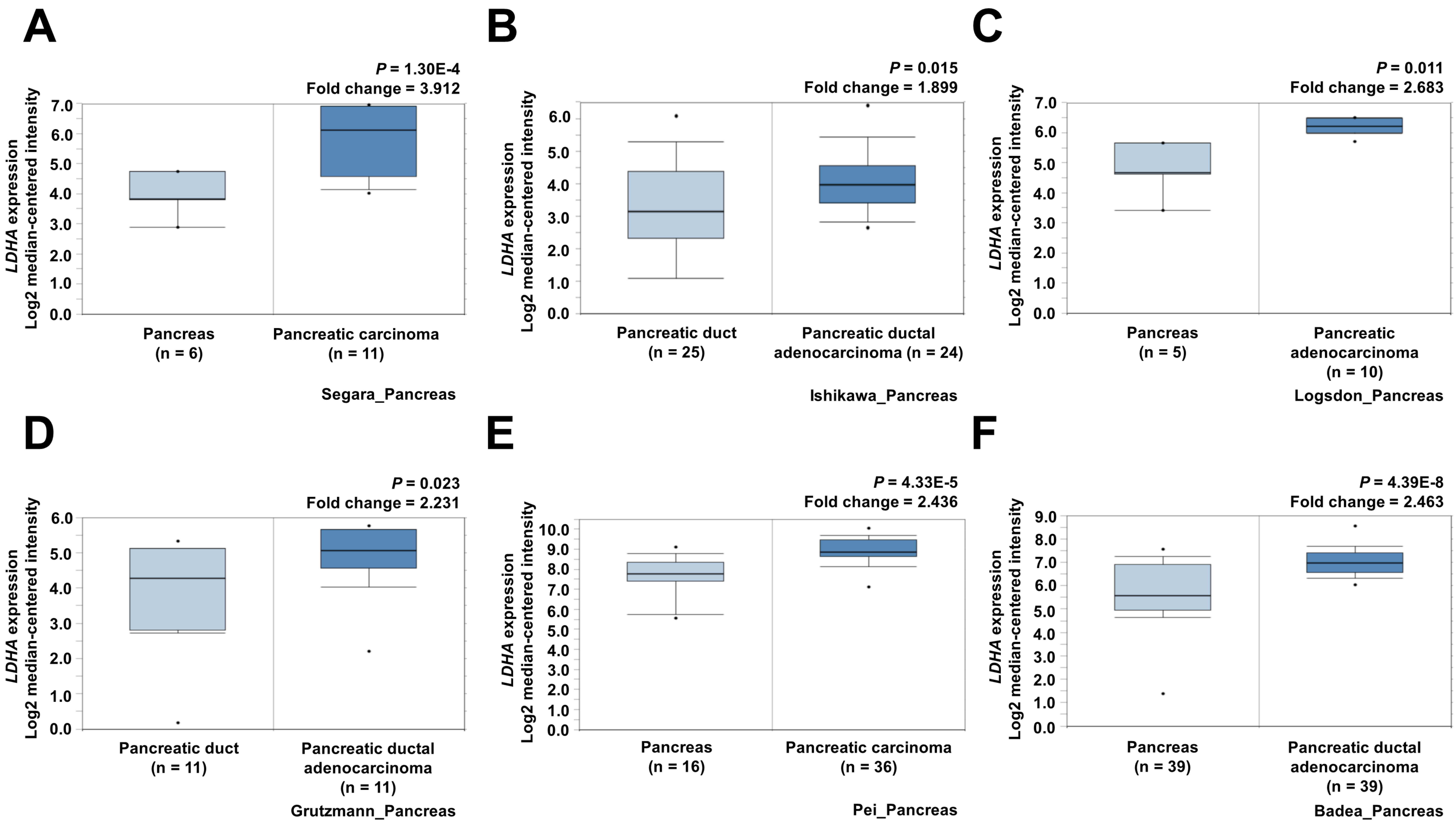

Supplementary Figure 5

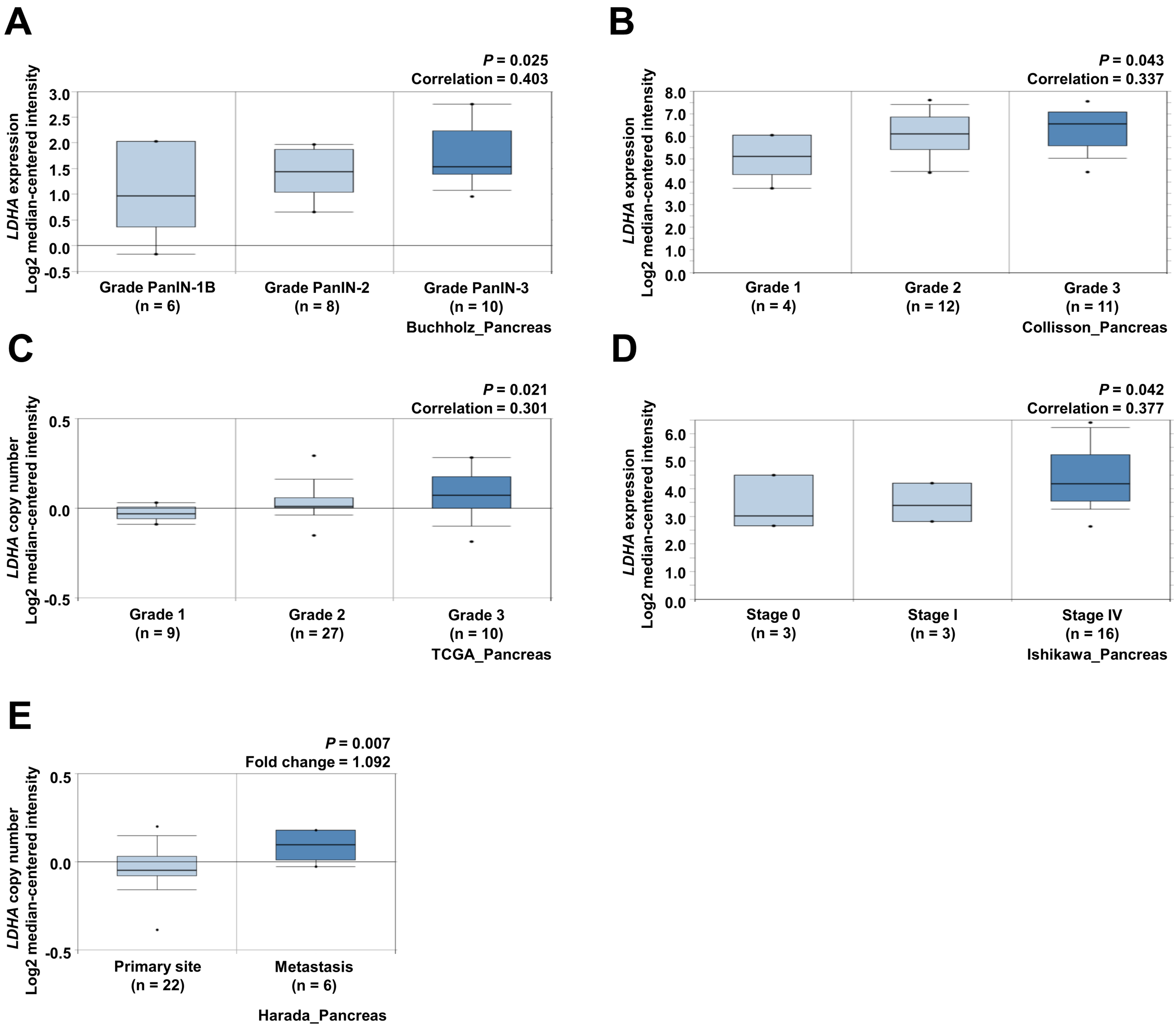

Supplementary Figure 6

A

Comparison: PANC-1/GEM with PANC-1 cells			
Up-regulated miRNA	Down-regulated miRNA		
hsa-miR-432-5p	hsa-miR-637		
hsa-miR-4497	hsa-miR-370		
hsa-miR-3960	hsa-miR-638		
hsa-miR-877-3p	hsa-miR-485-5p		
hsa-miR-3177-3p	hsa-miR-885-3p		
hsa-miR-31-5p	hsa-miR-3191-3p		
hsa-miR-5193	hsa-miR-3121-3p		
hsa-miR-6509-3p	hsa-miR-4314		
hsa-miR-449b-3p	hsa-miR-3150a-3p		
hsa-miR-2116-3p	hsa-miR-3151		
hsa-miR-6072	hsa-miR-3154		
hsa-miR-4487	hsa-miR-3192	hsa-miR-3192	
hsa-miR-4299	hsa-miR-4259	hsa-miR-4259	hsa-miR-4259
hsa-miR-4646-3p	hsa-miR-4286		
hsa-miR-1825	hsa-miR-3907		
hsa-miR-4436b-5p	hsa-miR-3135b		
hsa-miR-3173-5p	hsa-miR-4707-5p		
hsa-miR-3972	hsa-miR-4758-5p		
hsa-miR-4725-5p	hsa-miR-4776-5p		
hsa-miR-3918	hsa-miR-3660		
hsa-miR-1225-3p	hsa-miR-3919		
hsa-miR-3138	hsa-miR-4470	hsa-miR-4470	
hsa-miR-146a-5p	hsa-miR-4472		
hsa-miR-1224-3p	hsa-miR-4506		
hsa-miR-513a-5p	hsa-miR-4534		
hsa-miR-765	hsa-miR-4692		
hsa-miR-4653-3p	hsa-miR-4768-3	hsa-miR-4768-3p	
hsa-miR-4257	hsa-miR-4712-3p		
hsa-miR-4780	hsa-miR-5195-5p		
hsa-miR-4284	hsa-miR-6723-5p		
	hsa-miR-6083		

TargetScan (http://www.targetscan.org/)
miRanda (http://www.microrna.org/microrna/home.do)
DIANA-MICROT (http://www.microrna.gr/webServer)

B

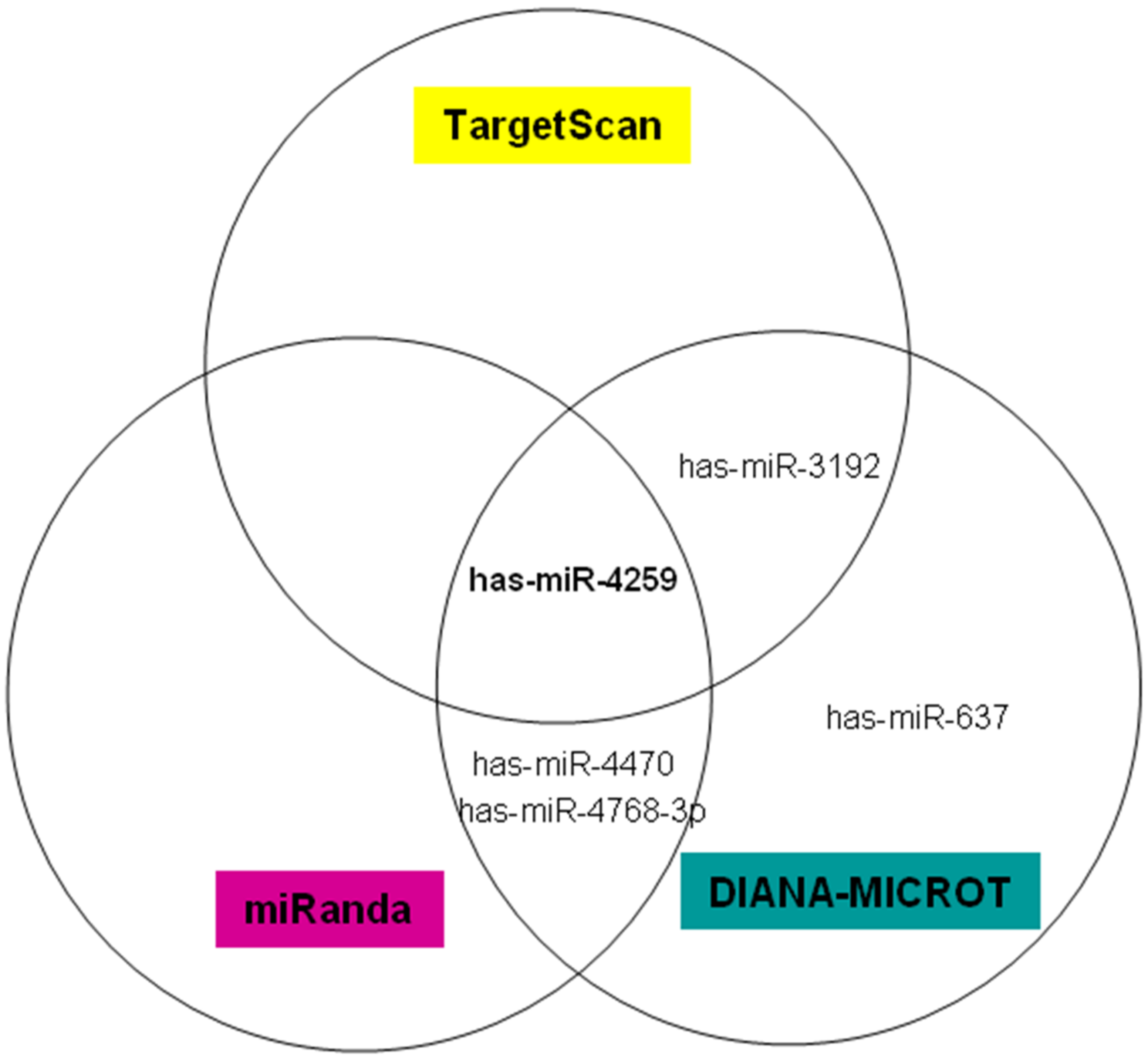

Supplementary Figure 7

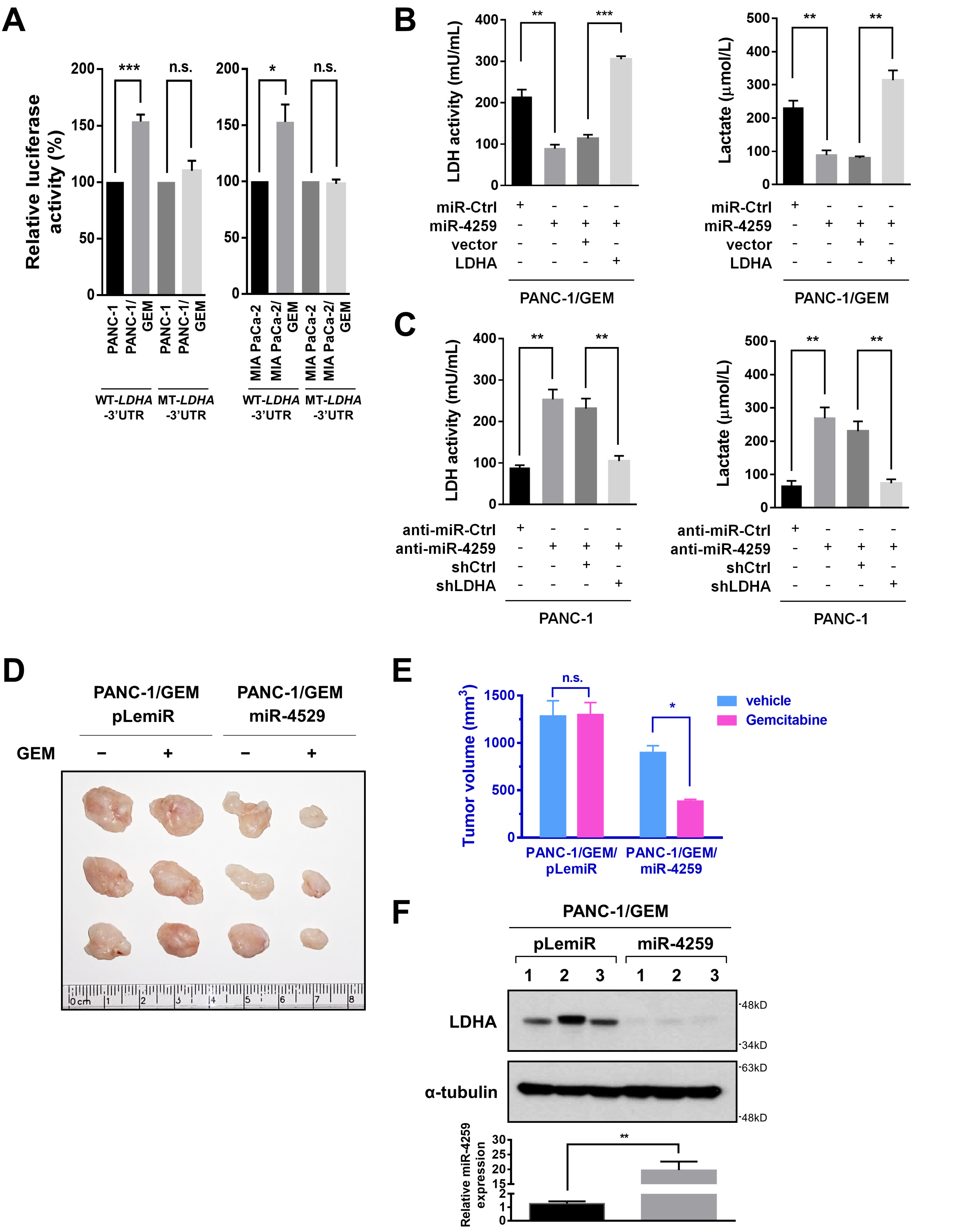

Supplementary Figure 8

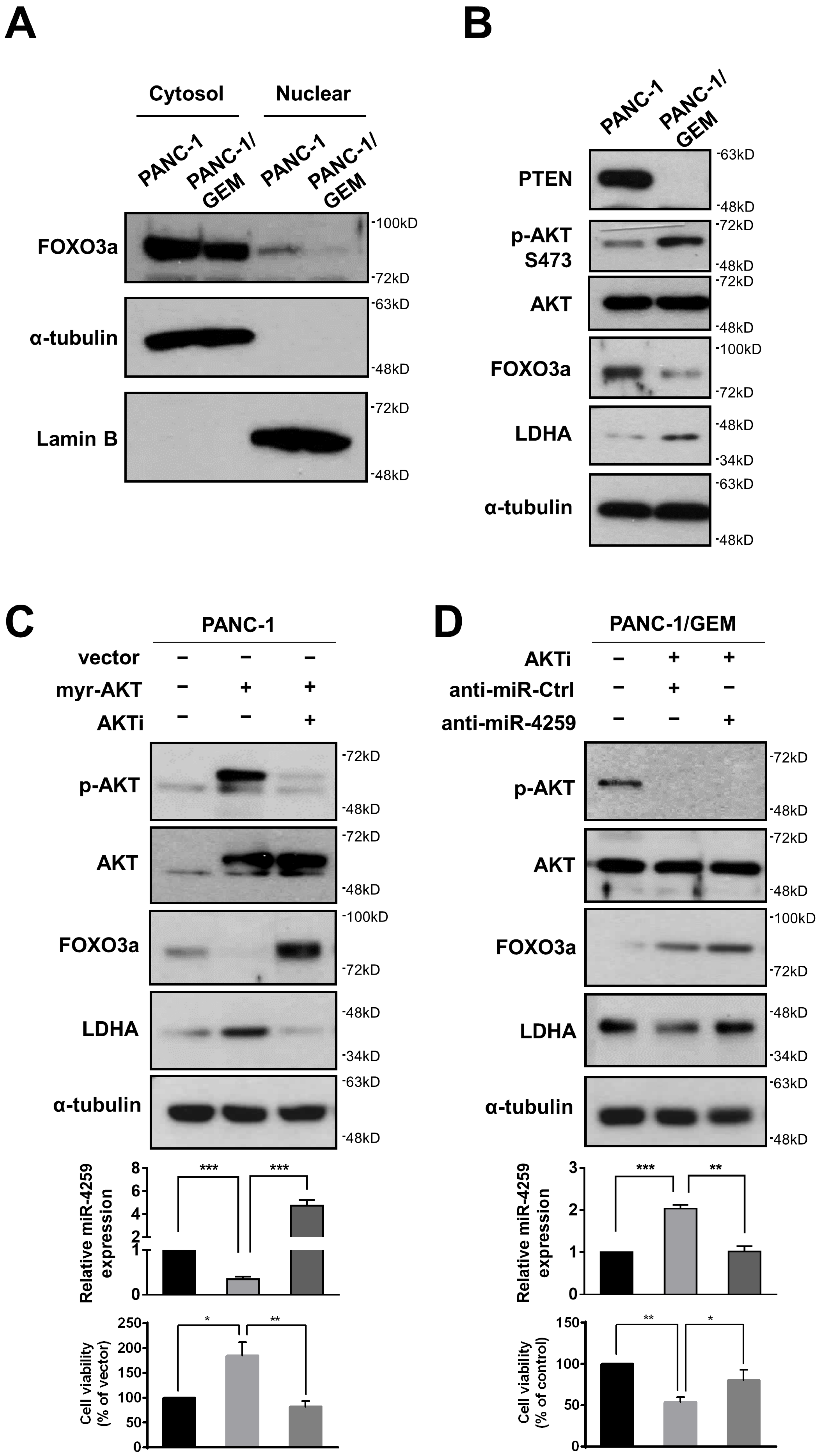

Supplementary Figure 9

A

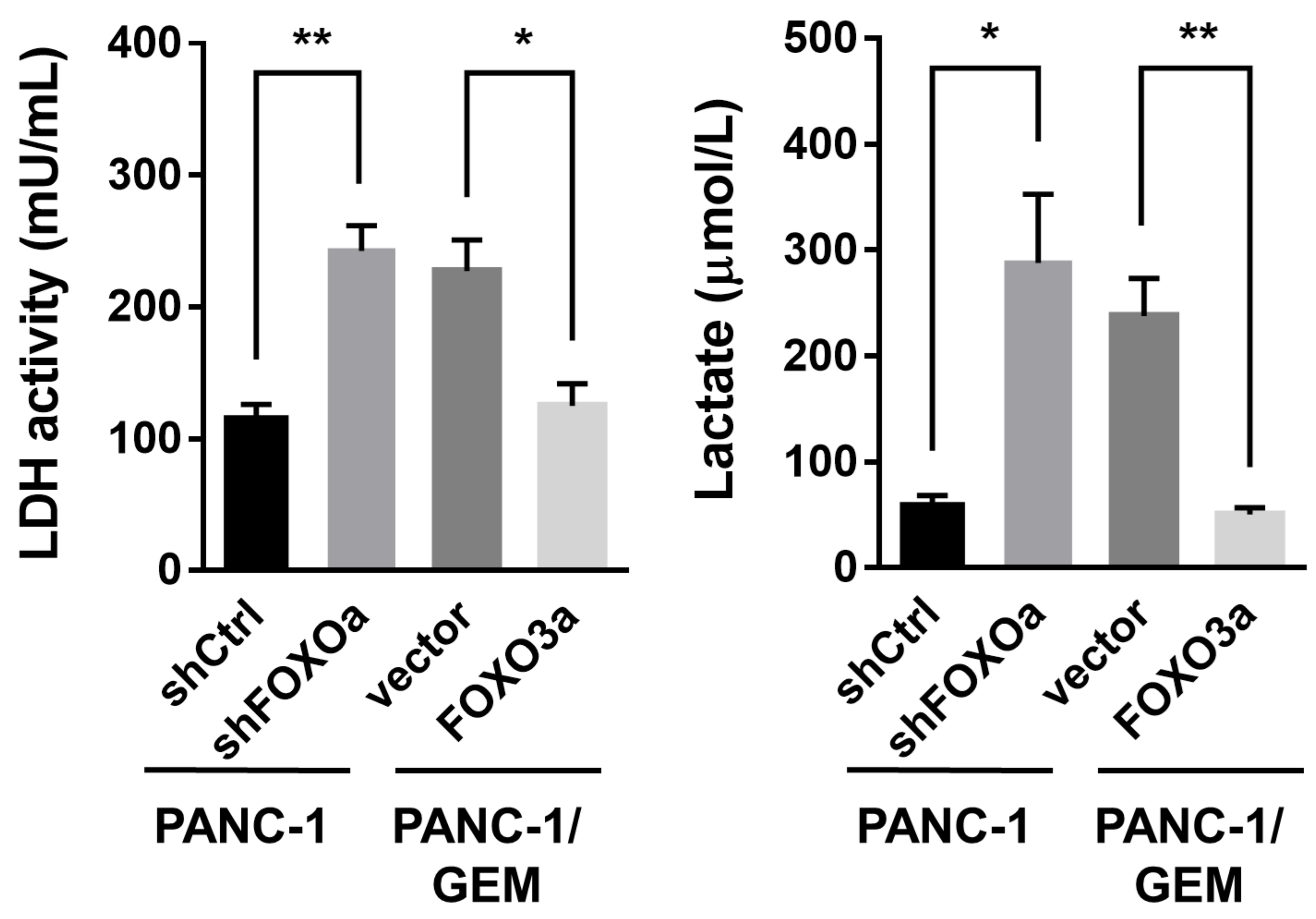

B

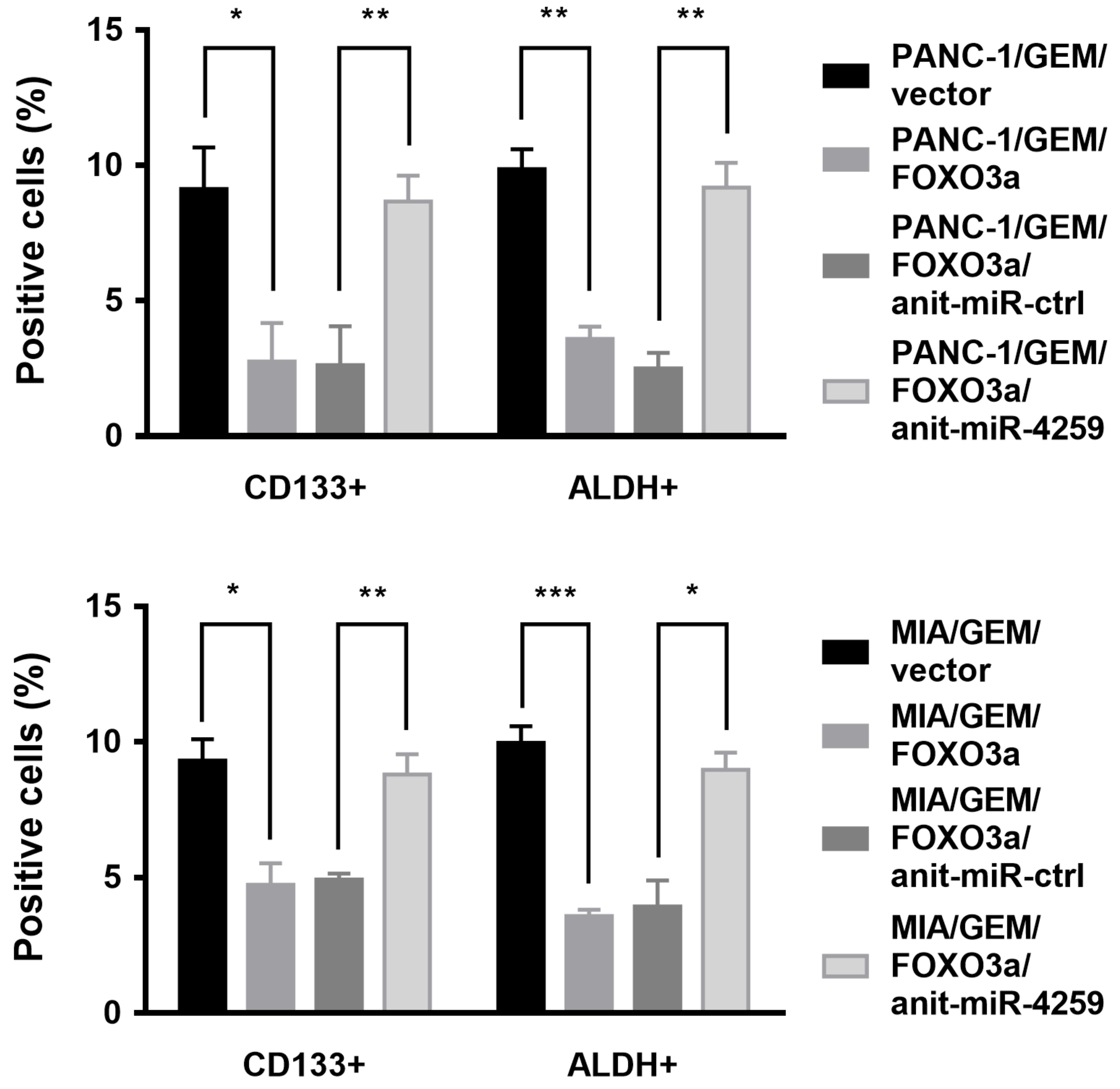

C

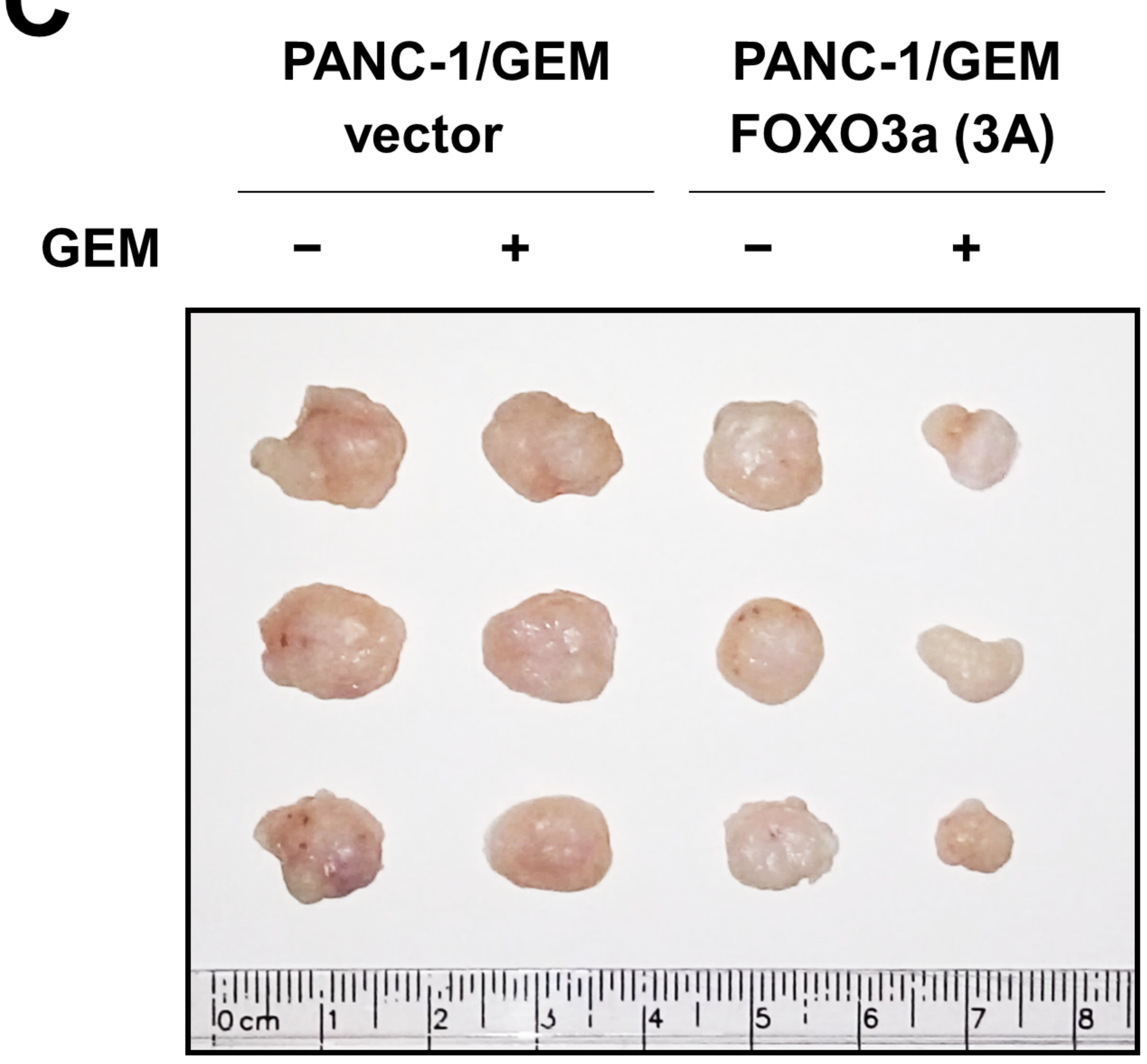

D

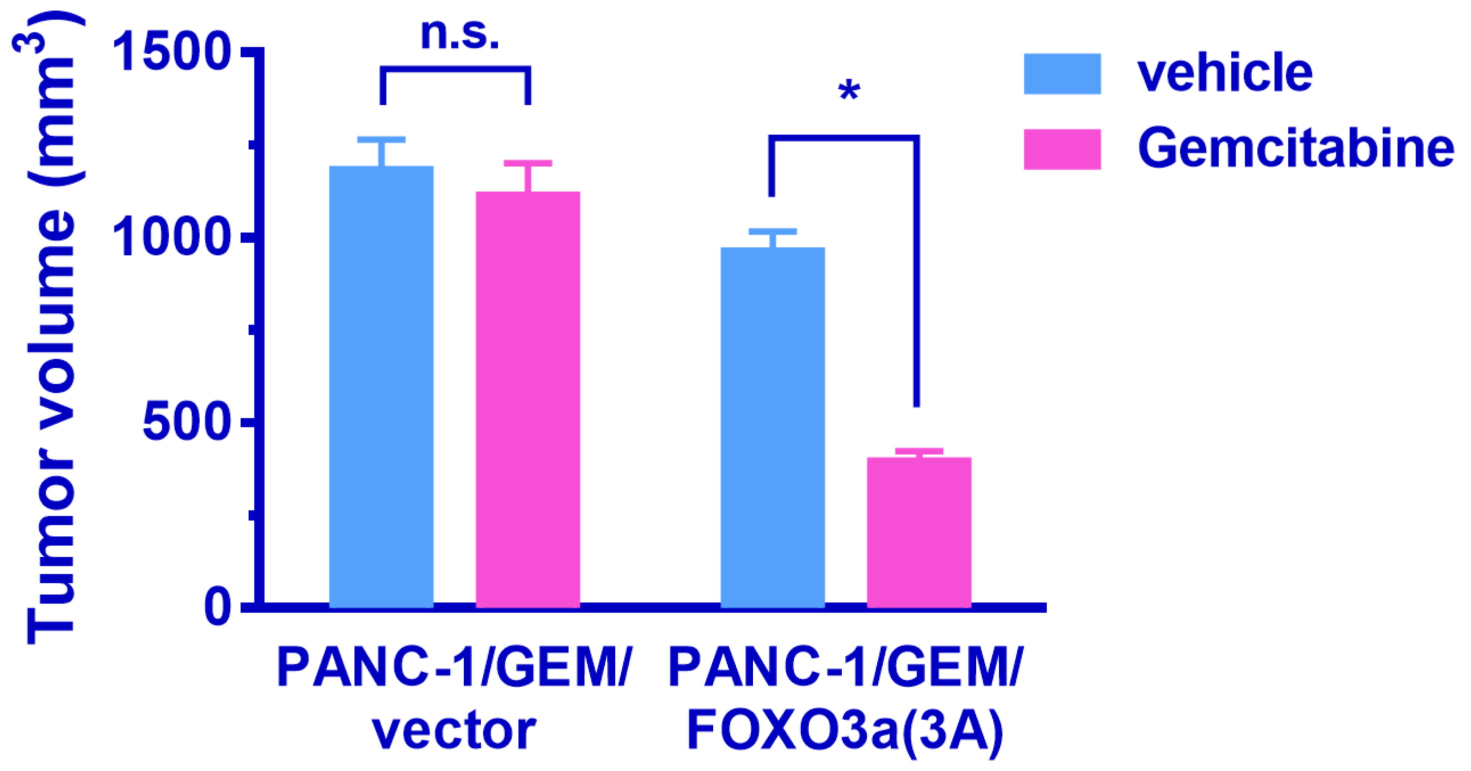

E

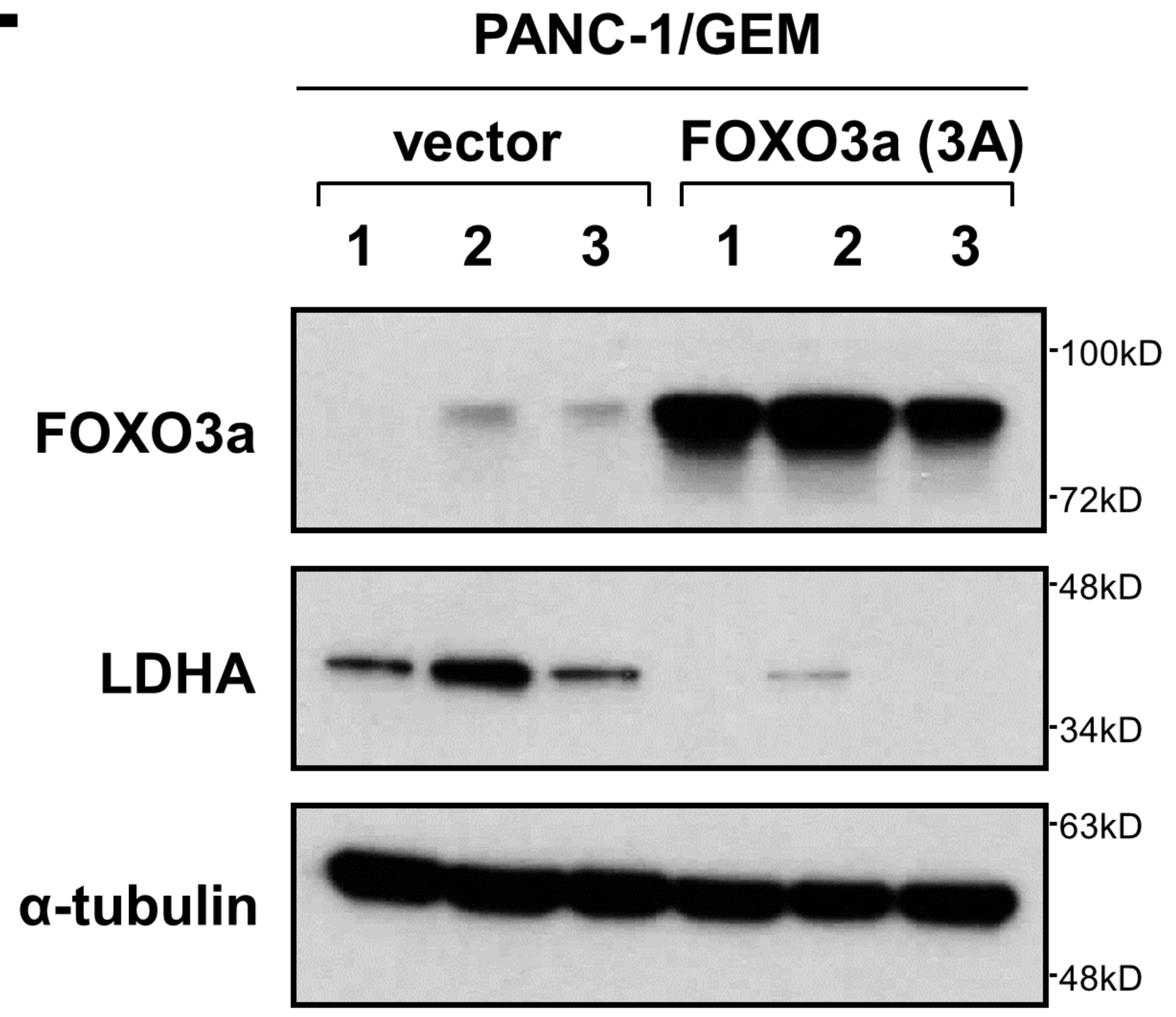

Supplement: Supplementary file 1 — Supplementary Material 1 [file 40170_2025_377_MOESM1_ESM.pdf]
