# Supplementary material for: FOXO3a/miR-4259-driven LDHA expression as a key mechanism of gemcitabine sensitivity in pancreatic ductal adenocarcinoma
Source: Cancer Metab. 2025 Feb 10;13:7. doi: 10.1186/s40170-025-00377-3 (PMC11809001; doi:10.1186/s40170-025-00377-3)
Supplement: Supplementary file 2 — Supplementary Material 2 [file 40170_2025_377_MOESM2_ESM.doc]

**Supplementary Tables**

Supplementary Table 1. The tumour-initiating cell frequency of PANC-1/GEM cells with knockdown of LDHA

| Cell number^*^  Group | 1,000 | 5,000 | 10,000 | 50,000 | TIC frequency^†^ | *P* value^‡^ |
| --- | --- | --- | --- | --- | --- | --- |
| PANC-1/GEM/  shCtrl | 1/5^#^ | 4/5 | 5/5 | 5/5 | 2,995 | 0.0021 |
| PANC-1/GEM/  shLDHA | 0/5 | 0/5 | 3/5 | 5/5 | 16,743 |

| ^*^The numbers of cells injected, ^#^numbers of mice and those positive for tumor growth (defined by tumour volume >100 mm^3^ in 49 days) are shown for control versus shLDHA-transduced PANC-1/GEM cells. ^†^Tumour-initiating cell (TIC) frequency and the ^‡^Chi-square test were calculated using the L-Calc software. |
| --- |

Supplementary Table 2. Association between cancer stem cell markers and *LDHA* expression in pancreatic cancer datasets

| **Stem cell markers** | **Datasets** | **Pearson correlation^†^, *r*** | ***P*-value^‡^** |  |
| --- | --- | --- | --- | --- |
| ***CD133*** | Pei (n = 52) | 0.3623 | 0.0083 |  |
|  | Segara (n = 17) | 0.5481 | 0.0227 |  |
| ***CD44*** | Badea (n = 78) | 0.7078 | < 0.0001 |  |
|  | Collisson (n = 27) | 0.5946 | 0.0011 |  |
|  | Grutzmann (n = 25) | 0.8386 | < 0.0001 |  |
|  | Pei (n = 52) | 0.5577 | < 0.0001 |  |
|  | Segara (n = 17) | 0.7184 | 0.0012 |  |
|  | TCGA (n = 75) | 0.6485 | < 0.0001 |  |
| ***SOX2*** | Grutzmann (n = 25) | 0.5025 | 0.0105 |  |
|  | TCGA (n = 75) | 0.3209 | 0.0050 |  |
| ***OCT4*** | Buchholz (n = 38) | 0.3316 | 0.0420 |  |
|  | Logsdon (n = 20) | 0.4792 | 0.0325 |  |
| ***KLF4*** | Badea (n = 78) | 0.6825 | < 0.0001 |  |
|  | Collisson (n = 27) | 0.4914 | 0.0092 |  |
|  | Grutzmann (n = 25) | 0.7337 | < 0.0001 |  |
|  | Iacobuzio-Donahue (n = 22) | 0.5238 | 0.0123 |  |
|  | Logsdon (n = 20) | 0.5448 | 0.0130 |  |
|  | Pei (n = 52) | 0.5284 | < 0.0001 |  |
|  | Segara (n = 17) | 0.7266 | 0.0010 |  |
| ***Nanog*** | Buchholz (n = 38) | 0.3792 | 0.0189 |  |
| ^†^*r*, Pearson's correlation coefficient and ^‡^*P*-value for two-tailed Student’s *t* test of Individual dataset (Oncomine database). | | | | |

Supplementary Table 3. The tumour-initiating cell frequency of PANC-1/GEM/miR-4259 cells with overexpressing LDHA

| Cell number^*^  Group | 1,000 | 5,000 | 10,000 | 50,000 | TIC frequency^†^ | *P* value^‡^ |
| --- | --- | --- | --- | --- | --- | --- |
| **PANC-1/GEM/**  **pLemiR** | 3/5^#^ | 4/5 | 4/5 | 5/5 | 3,404 | 0.0131^a^ |
| **PANC-1/GEM/**  **miR-4259** | 0/5 | 3/5 | 4/5 | 4/5 | 13,505 |
| **PANC-1/GEM/**  **miR-4259/vector** | 0/5 | 1/5 | 3/5 | 5/5 | 13,535 | 0.0250^b^ |
| **PANC-1/GEM/**  **miR-4259/LDHA** | 2/5 | 4/5 | 4/5 | 5/5 | 3,918 |

| ^*^The numbers of cells injected, ^#^numbers of mice and those positive for tumor growth (defined by tumour volume >100 mm^3^ in 49 days). ^†^Tumour-initiating cell (TIC) frequency and the ^‡^Chi-square test were calculated using the L-Calc software. ^a^PANC-1/GEM/miR-4259 group versus PANC-1/GEM/pLemiR group; ^b^PANC-1/GEM/miR-4259/LDHA group versus PANC-1/GEM/miR-4259/vector group. |
| --- |

Supplementary Table 4. The tumour-initiating cell frequency of PANC-1/GEM/FOXO3a(3A) cells with expression of miR-4259 antagomiR

| Cell number^*^  Group | 1,000 | 5,000 | 10,000 | 50,000 | TIC frequency^†^ | *P* value^‡^ |
| --- | --- | --- | --- | --- | --- | --- |
| **PANC-1/GEM/**  **vector** | 2/5^#^ | 3/5 | 5/5 | 5/5 | 3,384 | 0.0021^a^ |
| **PANC-1/GEM/**  **FOXO3a(3A)** | 0/5 | 2/5 | 3/5 | 4/5 | 18,718 |
| **PANC-1/GEM/**  **FOXO3a(3A)/**  **anti-miR-Ctrl** | 0/5 | 2/5 | 4/5 | 4/5 | 15,673 | 0.0310^b^ |
| **PANC-1/GEM/**  **FOXO3a(3A)/**  **anti-miR-4259** | 1/5 | 3/5 | 5/5 | 5/5 | 4,753 |

| ^*^The numbers of cells injected, ^#^numbers of mice and those positive for tumor growth (defined by tumour volume >100 mm^3^ in 49 days). ^†^Tumour-initiating cell (TIC) frequency and the ^‡^Chi-square test were calculated using the L-Calc software. ^a^PANC-1/GEM/FOXO3a(3A) group versus PANC-1/GEM/vector group; ^b^PANC-1/GEM/FOXO3a(3A)/anti-miR-4259 group versus PANC-1/GEM/FOXO3a(3A)/anti-miR-Ctrl group. |
| --- |

Supplementary Table 5. Association between *FOXO3* and *LDHA* expression in pancreatic cancer datasets

| ***FOXO3a* vs.** | **Datasets** | **Pearson correlation^†^, *r*** | ***P*-value^‡^** |
| --- | --- | --- | --- |
| ***LDHA*** | Buchholz (n = 38) | -0.5529 | 0.0003 |
|  | Grutzmann (n = 25) | -0.8266 | < 0.0001 |
|  | Ishikawa (n = 49) | -0.3463 | 0.0148 |
|  | Pei (n = 52) | -0.5364 | < 0.0001 |
|  | TCGA (n = 75) | -0.2717 | 0.0184 |
| ^†^*r*, Pearson's correlation coefficient and ^‡^*P*-value for two-tailed Student’s *t* test of Individual dataset (Oncomine database). | | | |

Supplementary Table 6. Association between cancer stem cell markers and *FOXO3a* expression in pancreatic cancer datasets

| **Stem cell markers** | **Datasets** | **Pearson correlation^†^, *r*** | ***P*-value^‡^** |  |
| --- | --- | --- | --- | --- |
| ***CD133*** | Collisson (n = 27) | -0.3838 | 0.0481 |  |
|  | Ishikawa (n = 49) | -0.2848 | 0.0473 |  |
| ***CD44*** | Badea (n = 78) | -0.4870 | < 0.0001 |  |
|  | Collisson (n = 27) | -0.5118 | 0.0063 |  |
|  | Grutzmann (n = 25) | -0.8541 | < 0.0001 |  |
|  | Ishikawa (n = 49) | -0.4405 | 0.0015 |  |
|  | Pei (n = 52) | -0.2919 | 0.0357 |  |
|  | TCGA (n = 75) | -0.3306 | 0.0038 |  |
| ***SOX2*** | Badea (n = 78) | -0.3999 | 0.0003 |  |
|  | Collisson (n = 27) | -0.5562 | 0.0026 |  |
|  | Grutzmann (n = 25) | -0.5495 | 0.0044 |  |
|  | TCGA (n = 75) | -0.4260 | 0.0001 |  |
| ***OCT4*** | Buchholz (n = 38) | -0.4092 | 0.0107 |  |
|  | Collisson (n = 27) | -0.6065 | 0.0008 |  |
|  | Ishikawa (n = 49) | -0.3103 | 0.0300 |  |
|  | Pei (n = 52) | -0.2873 | 0.0389 |  |
| ***KLF4*** | Collisson (n = 27) | -0.4144 | 0.0316 |  |
|  | Grutzmann (n = 25) | -0.7795 | < 0.0001 |  |
|  | Ishikawa (n = 49) | -0.3742 | 0.0081 |  |
|  | Pei (n = 52) | -0.4221 | 0.0018 |  |
|  | Segara (n = 17) | -0.6100 | 0.0093 |  |
| ***Nanog*** | Grutzmann (n = 25) | -0.4807 | 0.0150 |  |
| ^†^*r*, Pearson's correlation coefficient and ^‡^*P*-value for two-tailed Student’s *t* test of Individual dataset (Oncomine database). | | | | |

Supplementary Table 7. Sequences and information of PCR primers for mRNA, miRNA, ChIP and cloning

| **mRNA PCR primers** |
| --- |
| ***LDHA*** |
| 5’-GCAGATTTGGCAGAGAGTATAATG-3’ (forward) |
| 5’-GACATCATCCTTTATTCCGTAAAGA-3’ (reverse) |
| Universal ProbeLibrary Probe #31 |
| ***LDHB*** |
| 5’-GATGGATTTTGGGGGAACAT-3’ (forward) |
| 5’-AACACCTGCCACATTCACAC-3’ (reverse) |
| Universal ProbeLibrary Probe #5 |
| ***FOXO3a*** |
| 5’- cttcaaggataagggcgaca -3’ (forward) |
| 5’- cgactatgcagtgacaggttg -3’ (reverse) |
| Universal ProbeLibrary Probe #11 |
| ***SOX2*** |
| 5’-gggggaatggaccttgtatag-3’ (forward) |
| 5’-gcaaagctcctaccgtacca-3’ (reverse) |
| Universal ProbeLibrary Probe #65 |
| ***Nanog*** |
| 5’-ATGCCTCACACGGAGACTGT-3’ (forward) |
| 5’-CAGGGCTGTCCTGAATAAGC-3’ (reverse) |
| Universal ProbeLibrary Probe #69 |
| ***KLF4*** |
| 5’-GGGAGAAGACACTGCGTCA-3’ (forward) |
| 5’-GGAAGCACTGGGGGAAGT-3’ (reverse) |
| Universal ProbeLibrary Probe #52 |
| ***ALDHA1*** |
| 5’-CCAAAGACATTGATAAAGCCATAA-3’ (forward) |
| 5’-CACGCCATAGCAATTCACC-3’ (reverse) |
| Universal ProbeLibrary Probe #82 |
| ***CD133*** |
| 5’-GGAAACTAAGAAGTATGGGAGAACA-3’ (forward) |
| 5’-CGATGCCACTTTCTCACTGAT-3’ (reverse) |
| Universal ProbeLibrary Probe #86 |
| ***CD44*** |
| 5’-CAACAACACAAATGGCTGGT-3’ (forward) |
| 5’-CTGAGGTGTCTGTCTCTTTCATCT-3’ (reverse) |
| Universal ProbeLibrary Probe #40 |
| ***GAPDH*** |
| 5’-AGCCACATCGCTCAGACAC-3’ (forward) |
| 5’-GCCCAATACGACCAAATCC-3’ (reverse) |
| Universal ProbeLibrary Probe #60 |
|  |
| **Mature, pre- and pri-miR-4259 qRT-PCR primer** |
| ***miR-4259*** |
| 5’- GCGGCGGCAGTTGGGTCTAGGG -3’ (forward) |
| 5’- GTTGGCTCTGGTGCAGGGTCCGAGGTATTCGCACCAGAGCCAACTCCTGA -3’ (RT) |
| ***U47 (internal reference for micro-RNA)*** |
| 5’-CGGCGGTAATGATTCTGCCAAA-3’ (forward) |
| 5’-GTTGGCTCTGGTGCAGGGTCCGAGGTATTCGCACCAGAGCCAACACCTCAG-3’ (RT) |
| ***pre-miR-4259*** |
| 5’- TCTGAGTGGGGAAAGTGG -3’ (forward) |
| 5’- CCCAACTGTGACCTCCC -3’ (reverse) |
| ***pri-miR-4259*** |
| 5’- GCGGCAGAAATCCATTCA -3’ (forward) |
| 5’- AATTTGCCGCCTTCCTCTA -3’ (reverse) |
| ***GAPDH (internal reference for pre-miR-4259 and pri-miR-4259)*** |
| 5’-TGACCTGAAAGACCGACCAT-3’ (forward) |
| 5’-GTTCAGCCCCTCCCAGAC-3’ (reverse) |
| ***universal reverse primer for mature miRNAs*** |
| 5’-GTGCAGGGTCCGAGGT-3’ (reverse) |
|  |
| **Cloning primer** |
| ***LDHA*** |
| 5’- gggaattcATGGCAACTCTAAAGGATCA -3’ (forward) |
| 5’- gggcggccgcTCACTTGTCGTCATCGTCTTTGTAGTCAAATTGCAGCTCCTTTTG -3’ (reverse) |
| ***miR-4259*** |
| 5’- ggctcgagGCGGGTCAATCCTTCAGA -3’ (forward) |
| 5’- gggcggccgcCGATGCTATCCCTAATTCCTC -3’ (reverse) |
| ***FOXO3a*** |
| 5’-gggctagcATGGCAGAGGCACCGGCTT-3’ (forward) |
| 5’-gggcggccgcTCAGCCTGGCACCCAGCTCT-3’ (reverse) |
| ***LDHA-WT-3’UTR*** |
| 5’- gggctagcAAAAGGAGCTGCAATT -3’ (forward) |
| 5’- ggctcgagCAGTTGTATTTTCAGAAAAATG -3’ (reverse) |
| ***LDHA-MT-3’UTR (mutant 1, on 498)*** |
| 5’- ATACAAACAATGCAAC**GTTG**TATCCAAGTGTTATACCAAC -3’ (forward) |
| 5’- A**CAAC**GTTGCATTGTTTGTATGTAG -3’ (reverse) |
| ***LDHA-MT-3’UTR (mutant 2, on 518)*** |
| 5’- TATCCAAGTGTTATAC**GTTG**TAAAACCCCCAATAAACCTT -3’ (forward) |
| 5’- A**CAAC**GTATAACACTTGGATA -3’ (reverse) |
| ***LDHA-MT-3’UTR (mutant 3, on 818)*** |
| 5’- TGTAAAATTTATTTGC**GTTG**TGAATATAGGCAATGATAGT -3’ (forward) |
| 5’- A**CAAC**GCAAATAAATTTTACATTTA -3’ (reverse) |
|  |
| **miR-4259 promoter construct primer** |
| **miR-4529-promoter -2414~+43** |
| 5’- gggctagcGCCATTGCACACCTGATAGACT -3’ (forward) |
| 5’- ggctcgagCCCATCTGAGGTTCTCCCTC -3’ (reverse) |
| **miR-4529-promoter -1196~+43** |
| 5’- gggctagcGGCCTTCCCTTCTCCCTCTG -3’ (forward) |
| **miR-4529-promoter -612~+43** |
| 5’- gggctagcAAACTCTCCCAGGTGATTCTTA -3’ (forward) |
|  |
| ***FOXO3a BS* ChIP primer** |
| 5’- ATAGCCTTCAGTGCCCAAGT-3’ (forward) |
| 5’- CATGCTCCAAGCCTCACGAA-3’ (reverse) |
| **Nonsense control primer, NC** |
| 5’- TCAATGGCCCAACAAAGCAG-3’ (forward) |
| 5’- GTAGGTCTCAACAGTGGGCT-3’ (reverse) |
|  |
| ***FOXO3a BS* mutant** |
| 5’- TCACACCAAGAGGGGAGCTAATCAGCTAAT -3’ (forward) |
| 5’- ATTAGCTGATTAGCTCCCCTCTTGGTGTGA -3’ (reverse) |
|  |
| ***FOXO3a (3A)* mutant:** |
| ***FOXO3a 3A_*Thr32 to Ala (T32A) mutant** |
| 5’- CAGCTGCGTGCTGC**GCC**CTTGACGTTTCTC-3’ (forward) |
| 5’- GAGAAACGTCAAG**GGC**GCAGCACGCAGCTG-3’ (reverse) |
| ***FOXO3a 3A*_Ser253 to Ala (S253A) mutant** |
| 5’- GCGGAGCGGACGGCC**GCC**GCTGGTTCG-3’ (forward) |
| 5’- CGAACCAGCGGC**GGC**CGTCGCCGCTCCGC-3’ (reverse) |
| ***FOXO3a 3A_*Ser315 to Ala (S315A) mutant** |
| 5’- CAGCTGAGGGCGCC**GCC**CTGCGTCTG-3’ (forward) |
| 5’- CAGACGCAGGG**GGC**GGCGCCCTCAGCTG-3’ (reverse) |
|  |

The lowercase was representative to the additional sequence and restriction enzyme site was underlined. The mutation sequences were uppercase and underlined.
