# Supplementary material for: FOXO3a/miR-4259-driven LDHA expression as a key mechanism of gemcitabine sensitivity in pancreatic ductal adenocarcinoma
Source: Cancer Metab. 2025 Feb 10;13:7. doi: 10.1186/s40170-025-00377-3 (PMC11809001; doi:10.1186/s40170-025-00377-3)
Supplement: Supplementary file 3 — Supplementary Material 3 [file 40170_2025_377_MOESM3_ESM.doc]

Supplementary Figure 1. The gemcitabine sensitivity and cancer stem cell (CSC) properties of PDAC cells. (A) The PANC-1, PANC-1/GEM, MIA PaCa-2, MIA PaCa-2/GEM, BxPC-3 and SUIT-2 cell lines were treated with the indicated dosages of gemcitabine for 48 h and then analyzed cell viability by MTT assay and calculated the IC_50_. (B) The PDAC cell lines were treated with 4 μM gemcitabine (GEM) for 48 h and gemcitabine-induced cell death were measured by flow cytometric analysis. (C) Expression of *SOX2*, *KLF4* and *Nanog* were analyzed by RT-qPCR in PANC-1 and PANC-1/GEM cells (left), as well as in MIA PaCa-2 and MIA PaCa-2/GEM cells (right). (D) The positive cells of CD133 expression and ALDH activity of paired PANC-1 and paired MIA PaCa-2 cells were measured by flow cytometric analysis. Results are shown as means ± s.e.m. of three independent experiments. **P* < 0.05, ***P* < 0.01, ****P* < 0.001 and n.s. not significant (two-tailed Student’s *t* test).

Supplementary Figure 2. Metabolite analysis of PANC-1/GEM compared with PANC-1 cells by LC/MS. (A) The metabolites of PANC-1 and PANC-1/GEM cells were analyzed by LC/MS as described in Methods section and searched these m/z values by Biomolecules database of MarkerLynx software and latest version (version 3.6, online database) of The Human Metabolome Database (http://www.hmdb.ca/). *P* values <0.05 and a factor of change greater than 1.5 were selected for analysis. (B) Lactate production was measured among PANC-1, PANC-1/GEM, MIA PaCa-2 and MIA PaCa-2/GEM cells. The results are presented as the means ± s.e.m. of three independent experiments each performed in triplicate. ***P* < 0.01 and ****P* < 0.001 (two-tailed Student’s *t* test).

Supplementary Figure 3. LDHA expression associates with sphere formation and gemcitabine resistance of pancreatic cancer cells. (A) The *LDHA* and *LDHB* expression (left), and LDH enzyme activity (right) were determined between adherent and sphere types of SUIT-2 cells. (B) Left panel, the LDHA level (upper) and cell viability (bottom) of BxPC-3 cells with overexpressing LDHA were measured by Western blot and MTT assay after treatment with 2 μM of gemcitabine for 48 h. Right panel, The LDHA level (upper) and cell viability (bottom) of SUIT-2 cells with knockdown of LDHA were analyzed by Western blot and MTT assay after treatment with 2 μM of gemcitabine for 48 h. (C) The positive cells of CD133 expression and ALDH activity of the indicated MIA PaCa-2 cells were measured by flow cytometric analysis. (D) Expression of FOXO3a and LDHA were analyzed by Western blot, and miR-4259 expression was measured by RT-qPCR in BxPC-3, SUIT-2 PANC-1 and MIA PaCa-2 cells. Dot plot representing the CD133 expression (E) and ALDH activity (F) of the indicated cells were measured by flow cytometry analysis. The samples treated with IgG-PE were used as the control to set the gates defining the CD133-positive region and the specific ALDH inhibitor, diethylaminobenzaldehyde (DEAB) were used as the control to set the gates defining the ALDH-positive region. Results are presented as means ± s.e.m. of three independent experiments. **P* < 0.05, ***P* < 0.01 and ****P* < 0.001 (two-tailed Student’s *t* test).

Supplementary Figure 4. The association of *LDHA* expression between pancreas and pancreatic cancer. The *LDHA* expression positively correlates with pancreatic carcinoma. Oncomine datasets: Segara_Pancreas, Ishikawa_Pancreas, Logsdon_Pancreas, Grutzmann_Pancreas, Pei_Pancreas and Badea_Pancreas. Statistics from individual studies were obtained from the Oncomine database. The fold change (Log2 median-centered intensity) and *P*-value are shown within each box plot.

Supplementary Figure 5. The association of *LDHA* expression in advanced pancreatic cancer. The *LDHA* expression positively correlates with Grade PanIN, advanced stage and metastatic event in pancreatic carcinoma. Oncomine datasets: Buchholz_Pancreas, Ishikawa_Pancreas, Collisson_Pancreas, TCGA_Pancreas and Harada_Pancreas. Statistics from individual studies were obtained from the Oncomine database. The fold change (Log2 median-centered intensity) and *P*-value are shown within each box plot.

Supplementary Figure 6. miRNA microarray and on-line miRNAs database analysis between PANC-1/GEM and PANC-1 cells. (A) Analysis and comparison of PANC-1/GEM cells with PANC-1 cells by miRNA microarray were shown the up-regulated and down-regulated miRNAs as list. Colour square show the predicted miRNAs that tagets to *LDHA*-3’UTR form on-line databases. The putative miRNAs targeting to *LDHA*-3’UTR in miRanda (red square, 3 candidate miRNAs), TargetScan (yellow square, 2 candidate miRNA) and DIANA-MICROT (blue square, 5 candidate miRNAs). (B) A Venn diagram displaying the bioinformatic analyses of predicted miR-4259 candidate miRNAs target to *LDHA*-3’UTR.

Supplementary Figure 7. miR-4259 targets to *LDHA*3’UTR and regulates LDH activity, lactate production and gemcitabine resistance. (A) The luciferase reporter activity of the *LDHA*-3’UTR wild-type (WT-*LDHA*-3’UTR) and *LDHA*-3’UTR mutant reporters (MT-*LDHA*-3’UTR, triple-mutant sites, 498/518/818) in paired PANC-1 cells and paired MIA PaCa-2 cells. (B,C) The LDH activity (left) and lactate production (right) was measured in indicated cells. (D) Mice were subcutaneously implanted with the PANC-1/GEM/pLmiR and PANC-1/GEM/miR-4259 cells until the resulting tumours reached approximately 100 mm^3^. The mice were then intraperitoneally treated with vehicle or 50 mg/kg gemcitabine once a week (*n* = 5 per group). After twenty-four days, the tumours were dissected from the surrounding tissue of analysis. (E) Each column represents the means ± s.e.m. of the tumour volumes of five mice in each group. (F) LDHA expression was analyzed by Western blotting, and miR-4259 expression was measured by RT-qPCR in the indicated groups of tumour samples. α-tubulin was used as a loading control. The results are presented as the means ± s.e.m. **P* < 0.05, ***P* < 0.01, ****P* < 0.001 and n.s, not significant (two-tailed Student’s *t* test).

Supplementary Figure 8. The effects of AKT on FOXO3a/miR-4259/LDHA signaling. (A) Cytoplasmic and nuclear fractions from PANC-1 and PANC-1/GEM cells were assessed for the presence of FOXO3a, Lamin B (nuclear marker) and α-tubulin (cytosolic marker) by Western blot analysis. (B) Expression of PTEN, phosphor-AKT (S473), AKT, FOXO3a and LDHA were analyzed by Western blot between PANC-1 and PANC-1/GEM cells. α-tubulin was used as a loading control. (C) PANC-1 cells expressed myr-AKT (active form of Akt) were treated with AKT inhibitor (AKTi) and analyzed the expression of phosphor-AKT (S473), AKT, FOXO3a and LDHA by Western blot, as well as miR-4259 expression by RT-qPCR. (D) PANC-1/GEM cells expressed miR-4259 antagomiR (anti-miR-4259) were treated with 0.5 μM of AKTi and analyzed the expression of phosphor-AKT (S473), AKT, FOXO3a and LDHA by Western blot, miR-4259 expression by RT-qPCR, as well as measured cell viability by MTT assay. The results are presented as the means ± s.e.m. of three independent experiments. **P* < 0.05, ***P* < 0.01 and ****P* < 0.001 (two-tailed Student’s *t* test).

Supplementary Figure 9. FOXO3a regulates LDH activity, lactate production, CSC marker expression and gemcitabine resistance. (A) The LDH activity (left) and lactate production (right) were measured in the PANC-1 cells with knockdown of FOXO3a (shFOXO3a) and PANC-1/GEM cells with overexpression of FOXO3a. (B) The positive cells of CD133 expression and ALDH activity of indicated PANC-1/GEM cells (upper) and MIA PaCa-2/GEM (bottom) cells were measured by flow cytometric analysis. (C). Mice were subcutaneously implanted with the PANC-1/GEM/vector and PANC-1/GEM/FOXO3a (3A) cells until the resulting tumours reached approximately 100 mm^3^. The mice were then intraperitoneally treated with vehicle or 50 mg/kg gemcitabine once a week (*n* = 5 per group). After twenty-four days, the tumours were dissected from the surrounding tissue of analysis. (D) Each column represents the means ± s.e.m. of the tumour volumes of five mice in each group. (E) FOXO3a and LDHA expression were analyzed by Western blotting in the indicated groups of tumour samples. α-tubulin was used as a loading control. The results are presented as the means ± s.e.m. **P* < 0.05, ***P* < 0.01, ****P* < 0.001 and n.s, not significant (two-tailed Student’s *t* test).
